# ﻿Two new species of *Penicillium* and a new genus in *Xylariomycetidae* from the forest dump-sites in Chiang Mai, Thailand

**DOI:** 10.3897/mycokeys.116.150635

**Published:** 2025-04-29

**Authors:** Tanapol Thitla, Jutamart Monkai, Weiqian Meng, Surapong Khuna, Ning Xie, Sinang Hongsanan, Saisamorn Lumyong

**Affiliations:** 1 Shenzhen Key Laboratory of Microbial Genetic Engineering, College of Life Science and Oceanography, Shenzhen University, Shenzhen 518060, China; 2 Office of Research Administration, Chiang Mai University, Chiang Mai 50200, Thailand; 3 Department of Biology, Faculty of Science, Chiang Mai University, Chiang Mai 50200, Thailand; 4 Center of Excellence in Microbial Diversity and Sustainable Utilization, Chiang Mai University, Chiang Mai 50200, Thailand; 5 Academy of Science, The Royal Society of Thailand, Bangkok 10300, Thailand

**Keywords:** Eurotiomycetes, new taxa, *Pseudoleptodontidium*, soil fungi, Sordariomycetes, taxonomy

## Abstract

Waste accumulation in forest regions can have a severe impact on the soil mycobiome. However, research on soil fungi inhabiting forest disposal sites remains limited. Therefore, this study focused on the taxonomy and phylogeny of ascomycetes isolated from soil in forest dump-sites in Chiang Mai, Thailand. The fungal strains were identified using morphological characterisations and multigene phylogenetic reconstruction. A new genus, *Pseudoleptodontidium*, typified by *Ps.chiangmaiense***sp. nov.** (Amphisphaeriales genera *incertae sedis*, Xylariomycetidae), along with two new species, *Penicilliumchiangmaiense* (series *Janthinella*, section Lanata-Divaricata) and *P.terrae* (series *Erubescentia*, section Exilicaulis) (Aspergillaceae, Eurotiales), are described in detail and compared with closely-related species. Our discovery offers valuable insights into the soil ascomycetes associated with forest disturbances.

## ﻿Introduction

The disposal of waste materials through open burning, landfilling and dumping in land areas or water resources contributes to environmental issues, such as air pollution (PM_2.5_), as well as water and soil pollution, which can endanger the health and livelihood of humans, animals, plants and other organisms (Lin et al. 2020; Wanthongchai et al. 2021). Soil serves as a natural habitat for a wide range of fauna and flora, including fungi. Fungi are a major component of soil ecosystems, playing crucial roles in the cycling of nutrients and the decomposition of organic materials (Frąc et al. 2018; Coleine et al. 2022). The most abundant soil fungi belong to the Ascomycota, which includes the classes Arthoniomycetes, Dothideomycetes, Eurotiomycetes, Leotiomycetes and Sordariomycetes (Tedersoo et al. 2021; Gomes de Farias et al. 2023). Amongst these, *Fusarium*, *Penicillium* and *Phoma* are the most frequently isolated genera (Tedersoo et al. 2021; Yasanthika et al. 2023). However, contamination with pollutants may adversely affect their diversity, population and ecological functions (Frąc et al. 2018; Schloter et al. 2018; Coleine et al. 2022). The ability to synthesise a wide range of enzymes for breaking down various substrates enables soil fungi to adapt and thrive in diverse environments and harsh conditions (Singh et al. 2021; Coleine et al. 2022; Sun et al. 2024).

Extensive studies have focused on isolating and characterising soil fungi from contaminated areas, landfills and urban dump-sites (Sangale et al. 2019; Verma and Gupta 2019; Ren et al. 2021; Khan et al. 2022; Gong et al. 2023; Sathiyabama et al. 2024; Sun et al. 2024). These studies have revealed diverse soil fungal communities and identified numerous new fungal taxa and strains from these polluted habitats. Moreover, they have demonstrated a significant potential for biodegradation and bioremediation. For example, Yasanthika et al. (2021) studied soil ascomycetes in China and reported a new species, *Juxtiphomayunnanensis*, as well as two new records, *Lecanicilliumdimorphum* and *Scopulariopsisbrevicaulis*, from urban-industrialised soils. Ren et al. (2021) isolated 29 fungal strains from soils contaminated with explosive materials in China. Amongst them, the isolate of *Fusariumsolani* demonstrated the ability to decompose alkyne-terminated polybutadiene with urethane segments (PUPB) (Ren et al. 2021). Similarly, Sangale et al. (2019) obtained 109 fungal isolates from the dumping sites of mangrove rhizosphere soil and revealed that the strains of *Aspergillusterreus* and *A.sydowii* were the most effective in breaking down polythene. Additionally, the strain of *Penicilliumcitrinum*, isolated from municipal landfill soils in Bhopal, India, has demonstrated efficacy in degrading low-density polyethylene (LDPE) without prior pretreatment (Khan et al. 2022).

Dump-sites, especially those located within forested areas, represent an underexplored yet ecologically significant niche. Forest dump-sites provide a distinctive habitat, characterised by decreased soil nutrients, fluctuating temperature and moisture levels and potential exposure to pollutants (Kooch et al. 2023; Sun et al. 2024). It is essential for exploring novel soil fungi from this habitat in order to determine fungal diversity and investigate their biodegradation strategies. Therefore, the present study aims to isolate and identify soil ascomycetes from disposal sites located in forests of northern Thailand. The topsoil samples from forest dump-sites in Chiang Mai Province were collected and isolated for fungi, leading to the discovery of five novel Ascomycota strains. Based on molecular analyses and morphological characteristics, two new species of *Penicillium* and a new genus in Xylariomycetidae were introduced and described.

## ﻿Materials and methods

### ﻿Fungal isolation

**Figure 1.**
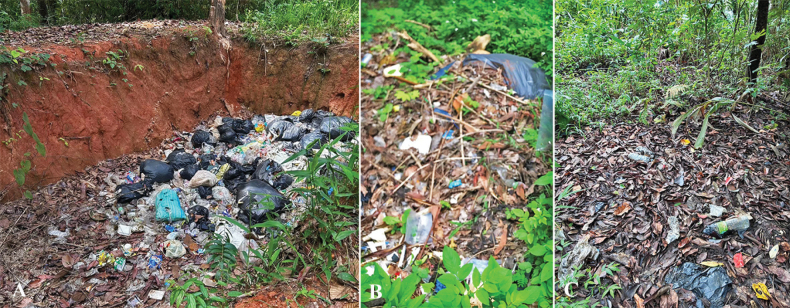
Forest dump-sites used for soil fungal isolation in this study **A** Papae, Mae Tang District, Chiang Mai Province **B** Suthep, Muang Chiang Mai District, Chiang Mai Province **C** Mae Sa, Mae Rim District, Chiang Mai Province.

Soil samples (0–10 cm depth) were collected from three forest dump-sites in June 2024 in Chiang Mai Province, Thailand: (1) Papae, Mae Taeng District, (2) Suthep, Muang Chiang Mai District and (3) Mae Sa, Mae Rim District (Fig. 1). The collection details were noted (Rathnayaka et al. 2024) and the soil samples were placed in plastic bags and taken to the Sustainable Development of Biological Resources Laboratory (SDBR), at the Department of Biology, Faculty of Science, Chiang Mai University, Thailand. Upon arrival, soil fungi were isolated immediately using the serial dilution plating method with three serial dilutions in sterile water (Yasanthika et al. 2022). After dilution, 100 µl of the soil suspension was dropped and spread on potato dextrose agar (PDA; CONDALAB, Spain) supplemented with 100 µg/ml of streptomycin. The isolation plates were incubated at 25 °C in the dark for 5 days. The appearing fungal colonies were transferred to fresh PDA using the hyphal tip method (Korhonen and Hintikka 1980). The pure cultures were deposited and permanently preserved in a metabolically inactive state at the Culture Collection of Microbial Shenzhen University (MBSZU), Shenzhen University, China.

### ﻿Morphological characterisation

The morphological characteristics of the obtained fungi were observed in both macro-morphology and micro-morphology, with different details depending on each fungus.

To investigate the morphology of *Penicillium* (comprising MBSZU 24-007 to MBSZU 24-010), the colony characteristics, growth rate, pigment production, sporulation or related features were investigated on Blakeslee’s Malt extract agar (MEAbl), creatine sucrose agar (CREA), Czapek yeast autolysate agar with 5% NaCl (CYAS), Czapek’s agar (CZ), Dichloran 18% glycerol agar (DG18), malt extract agar (MEA), oatmeal agar (OA), PDA and yeast extract sucrose agar (YES) at 25 °C in darkness for 7 days. The experiment was also performed on Czapek yeast autolysate agar (CYA) at 25, 30 and 37 °C in darkness for 7 days to characterise the macro-morphology (Visagie et al. 2014; Khuna et al. 2023). Micro-morphologically, the characteristics of conidiophores, stipes, conidiogenous cells, conidia or other structures were observed under a light microscope (Nikon DS-Ri2; Nikon, Japan), using fungal colonies grown on MEA at 25 °C in darkness for 7 days. Size data were evaluated by at least 50 measurements per structure.

The colony characteristics, growth rate and pigment production of *Pseudoleptodontidium* (MBSZU 25-005) were studied on PDA and MEA at 25 °C in darkness for 14 days. Micro-morphology was observed under a light microscope using a fungal colony grown on PDA at 25 °C in darkness for 14–21 days. The size of each morphological structure was measured at least 50 times per structure.

### ﻿DNA extraction, amplification and sequencing

Fungal genomic DNA from each strain was extracted from the fungal mycelium, which had grown on PDA at 25 °C for a week, using an E.Z.N.A^®^ Tissue DNA Kit (Omega, USA). The polymerase chain reaction (PCR) technique was used to amplify each region. Each target locus was amplified using the specific primers (Table 1). PCR amplifications were performed in 20 µl reaction mixtures, consisting of 1 µl of genomic DNA, 1 µl of each primer, 10 µl of 2× Phanta Max Master Mix (Dye Plus) (Vazyme, China) and 7 µl of deionised water. The PCR amplification was performed using a T100 Thermal Cycler (BIO-RAD, USA), with an initial denaturation step at 95 °C for 5 min, followed by 35 cycles of denaturation, annealing and elongation steps. The denaturation and elongation steps were performed at 95 °C for 30 s and 72 °C for 60 s, respectively. The annealing step was performed at different conditions depending on each target locus (Table 1). A final elongation step was performed at 72 °C for 10 minutes. The success or failure of the PCR product was determined through 1% agarose gel electrophoresis, followed by purification of the product using the E.Z.N.A^®^ Gel Extraction Kit (Omega, USA). The quality and quantity of the purified PCR products were assessed using 1% agarose gel electrophoresis and a Nanodrop 2000 Spectrophotometer (Thermo Scientific, USA). Subsequently, the products were sequenced by BGI-Shenzhen Company (Shenzhen, Guangdong, China).

**Table 1.** The specific primer and annealing condition of each locus used in this study.

Loci*	Primer	Annealing condition		References
		Temperatures (°C)	Annealing period (s)	
ITS	ITS4/ITS5	52	30	White et al. (1990)
LSU	LR0R/LR5	52	30	Vilgalys and Hester (1990); Rehner and Samuels (1994)
*CAM*	CF1/CF4	51	60	Peterson et al. (2005)
	Cmd5/Cmd6	58	30	Hong et al. (2006)
*RPB2*	fRPB2-5F/ fRPB2-7cR	56	60	Liu et al. (1999)
*TUB*	Bt2a/Bt2b	52	30	Glass and Donaldson (1995)
	T1/Bt2b	55	45	Glass and Donaldson (1995); O’Donnell and Cigelnik (1997)

* ITS – Internal Transcribed Spacer region of the rRNA; LSU – 28S large subunit of the nuclear rRNA; *TUB* – beta-tubulin gene; *CAM* – calmodulin gene; *RPB2* – RNA polymerase II second largest subunit genes.

The bidirectional sequence data were assembled using the software Sequencher 5.4.6 (Nishimura 2000). The consensus sequence data were searched for sequence similarity via the Basic Local Alignment Search Tool (BLAST) in the National Center for Biotechnology Information (NCBI) website.

### ﻿Phylogenetic analysis

The multi-loci phylogenetic dataset was obtained, based on previous studies of PenicilliumsectionExilicaulis (Ansari et al. 2023; Liu et al. 2023; Wang et al. 2023b; Visagie et al. 2024a, 2024b), PenicilliumsectionLanata-Divaricata (Lenz et al. 2022; Liu et al. 2023; Wang et al. 2023b; Araújo et al. 2024; Visagie et al. 2024b) and Xylariomycetidae (Samarakoon et al. 2022; Crous et al. 2023; Li et al. 2024; Samarakoon 2024) (Suppl. material 1: tables S1–S3). The sequence data for each locus were individually aligned using MUSCLE through the software MEGA 6 (Edgar 2004) and manually adjusted in BioEdit v.7.2.5 (Hall 2004). The concatenation of the ITS, *TUB*, *CAM* and *RPB2* loci was performed for the phylogenetic analysis of *Penicillium*; in contrast, the combined ITS, LSU, *RPB2* and *TUB* loci were used for the analysis of Xylariomycetidae. Maximum Likelihood (ML) and Bayesian Inference (BI) analyses were applied to generate a phylogenetic tree. The ML analysis was conducted with 25 categories and 1,000 bootstrap (BS) replications under the GTRCAT model using RAxML-HPC2 on XSEDE (v.8.2.12) in the CIPRES web portal (Felsenstein 1985; Stamatakis 2006; Miller et al. 2009). The best-fit models of nucleotide substitution for individual locus were determined by using MrModelTest v.2.3 based on the Akaike Information Criterion (AIC) (Nylander 2004). The GTR+I+G substitution model was the best fit for all loci. The BI analysis was performed using MrBayes v.3.2.6 (Ronquist and Huelsenbeck 2003). Bayesian Posterior Probability (PP) was examined by Markov Chain Monte Carlo (MCMC) sampling. Six simultaneous Markov chains were run with random initial trees, wherein every 100^th^ generation was sampled. The first 20% of generated trees, representing the burn-in phase of the analysis, were discarded, while the remaining trees were used to calculate PP in the majority-rule consensus tree. The tree topologies were visualised in FigTree v.1.4.0 with BS support and PP values equal to or higher than 75% and 0.95, respectively, in branches (Rambaut 2019). The final alignment and phylogram were submitted to TreeBASE (http://purl.org/phylo/treebase/phylows/study/TB2:S32075, accessed 19 March 2025).

## ﻿Results

### ﻿Phylogenetic analysis

Phylogenetic analysis of 72 taxa from *Penicillium*, section Exilicaulis (including *P.terrae*MBSZU 24-007 and MBSZU 24-008) was performed using a combined ITS, *TUB*, *CAM* and *RPB2* sequence dataset. *Penicilliumjanthinellum* CBS 340.48 and *P.limosum* CBS 339.97 were selected as the outgroup. The combined dataset comprised 2,630 characters (ITS, 1−564 bp; *TUB*, 565−1,102 bp; *CAM*, 1,103−1,701 bp; *RPB2*, 1,702−2,630 bp), including gaps. RAxML analysis of the integrated dataset yielded the best-scoring tree with a final ML optimisation likelihood value of -26380.0905. The matrix contained 1,279 distinct alignment patterns, with 13.06% of the characters being undetermined or gaps. The estimated base frequencies were recorded as follows: A = 0.2238, C = 0.2765, G = 0.2706 and T = 0.2291, while the substitution rates were as follows: AC = 1.0947, AG = 3.5202, AT = 1.1705, CG = 0.7818, CT = 5.4306 and GT = 1.0000. The gamma distribution shape parameter alpha value was equal to 0.2342, while the tree length was equal to 2.4771. The final average standard deviation of the split frequencies at the end of the total MCMC generations was computed as 0.003644 via BI analysis.

Phylogenetic analysis of 111 taxa from PenicilliumsectionLanata-Divaricata (including *P.chiangmaiense*MBSZU 24-009 and MBSZU 24-010) was performed using a combined ITS, *TUB*, *CAM* and *RPB2* sequence dataset. *Penicilliumalogum* CBS 140996 and *P.stolkiae* CBS 315.67 were selected as outgroups. The combined dataset comprised 2,549 characters (ITS, 1−563 bp; *TUB*, 564−1,114 bp; *CAM*, 1,115−1,794 bp; *RPB2*, 1,795−2,549 bp), including gaps. RAxML analysis of the integrated dataset yielded the best scoring tree with a final ML optimisation likelihood value of -35195.9174. The matrix contained 1,381 distinct alignment patterns with 12.17% undetermined characters or gaps. The estimated base frequencies were recorded as follows: A = 0.2214, C = 0.2908, G = 0.2615 and T = 0.2263, while the substitution rates were as follows: AC = 1.1361, AG = 3.5568, AT = 1.5061, CG = 0.7521, CT = 5.3860 and GT = 1.0000. The gamma distribution shape parameter alpha value was equal to 0.2744, while the tree length was equal to 3.5928. The final average standard deviation of the split frequencies at the end of the total MCMC generations was computed as 0.005628 via BI analysis.

Phylogenetic analysis of species in subclass Xylariomycetidae was performed using a combined ITS, LSU, *RPB2* and *TUB* sequence dataset of MBSZU 25-005 (proposed as *Pseudoleptodontidiumchiangmaiensis*), together with 118 taxa of the subclass. *Achaetomiummacrosporum* CBS 532.94, *Chaetomiumelatum* CBS 374.66 and *Sordariafimicola* CBS 723.96 were selected as outgroups. The combined dataset comprised 3,560 characters (ITS, 1−693 bp; LSU, 694−1,592 bp; *RPB2*, 1,593−2,656 bp; *TUB*, 2,657−3,560 bp), including gaps. RAxML analysis of the integrated dataset yielded the best scoring tree with a final ML optimisation likelihood value of -83630.121273. The matrix contained 2,615 distinct alignment patterns with 39.42% undetermined characters or gaps. The estimated base frequencies were recorded as follows: A = 0.256414, C = 0.231937, G = 0.280501 and T = 0.231149, while the substitution rates were as follows: AC = 0.888171, AG = 2.661198, AT = 1.161270, CG = 0.868099, CT = 3.494813 and GT = 1.000000. The gamma distribution shape parameter alpha value was equal to 0.351763, while the tree length was equal to 15.592567. The final average standard deviation of the split frequencies at the end of the total MCMC generations was computed as 0.009989 via BI analysis.

**Figure 2.**
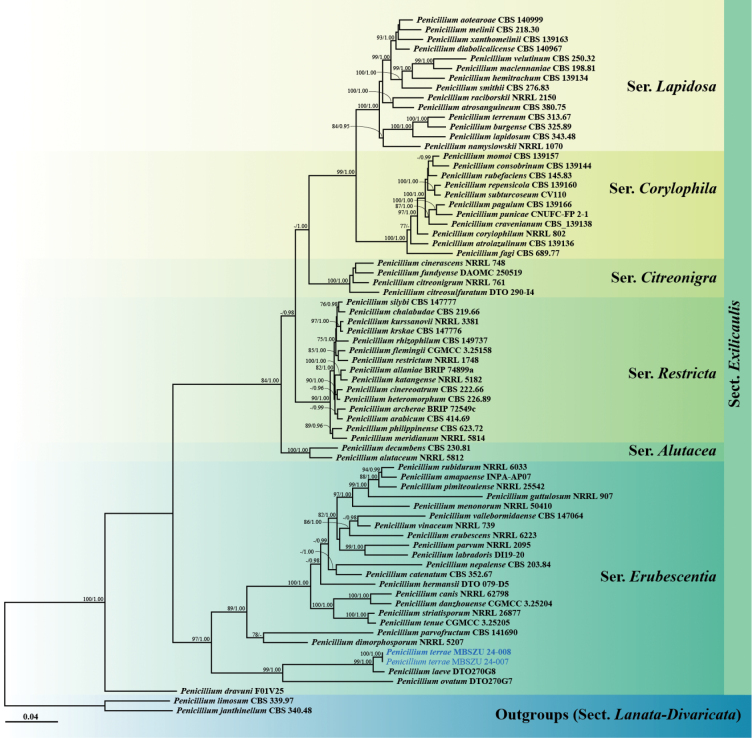
Phylogram generated from Maximum Likelihood analysis of 72 specimens belonging to the genus PenicilliumsectionExilicaulis, using the combined ITS, *TUB*, *CAM* and *RPB2* genes. *Penicilliumjanthinellum* CBS 340.48 and *P.limosum* CBS 339.97 were used as the outgroup. The numbers above branches show bootstrap percentages (left) and Bayesian Posterior Probabilities (right). Bootstrap values ≥ 75% and Bayesian Posterior Probabilities ≥ 0.95 are shown. The scale bar reflects the estimated number of nucleotide substitutions per site. The fungal strains in this study are blue. Type species are bold.

Topologically, the ML and BI phylogenetic trees of all fungal species had similar results; therefore, only the ML phylogram was demonstrated in this study. The phylogram of PenicilliumsectionExilicaulis showed that two new strains (MBSZU 24-007 and MBSZU 24-008) separated from other recognised species with 100% BS and 1.00 PP supports (Fig. 2). These fungal strains formed a sister clade with *P.laeve* DTO270G8 (BS 99% and PP 1.00) and belonged to the series *Erubescentia*.

**Figure 3.**
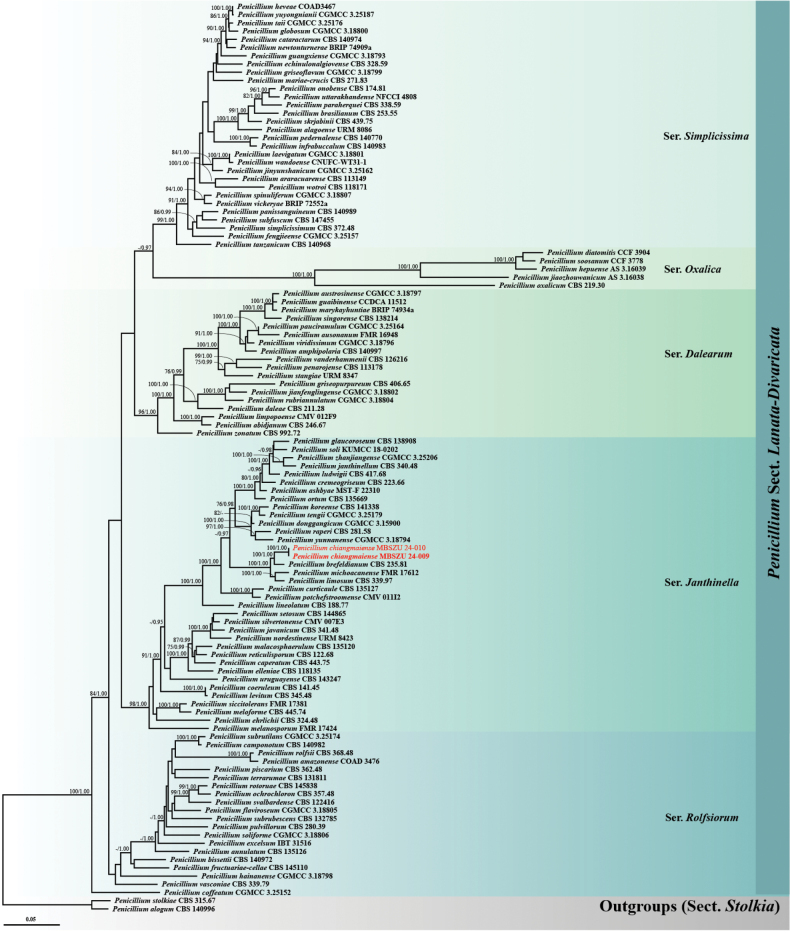
Phylogram generated from Maximum Likelihood analysis of 111 specimens belonging to the genus PenicilliumsectionLanata-Divaricata using the combined ITS, *TUB*, *CAM* and *RPB2* genes. *Penicilliumalogum* CBS 140996 and *P.stolkiae* CBS 315.67 were used as the outgroup. The numbers above branches show bootstrap percentages (left) and Bayesian Posterior Probabilities (right). Bootstrap values ≥ 75% and Bayesian Posterior Probabilities ≥ 0.95 are shown. The scale bar reflects the estimated number of nucleotide substitutions per site. The fungal strains in this study are red. Type species are bold.

While the phylogram of PenicilliumsectionLanata-Divaricata exhibited that MBSZU 24-009 and MBSZU 24-010 formed a distinct clade, clearly separated from other taxa with significant support (BS 100% and PP 1.00; Fig. 3). These strains also formed a sister clade with *P.brefeldianum* CBS 235.81 (BS 100% and PP 1.00) within the Series *Janthinella* clade.

**Figure 4.**
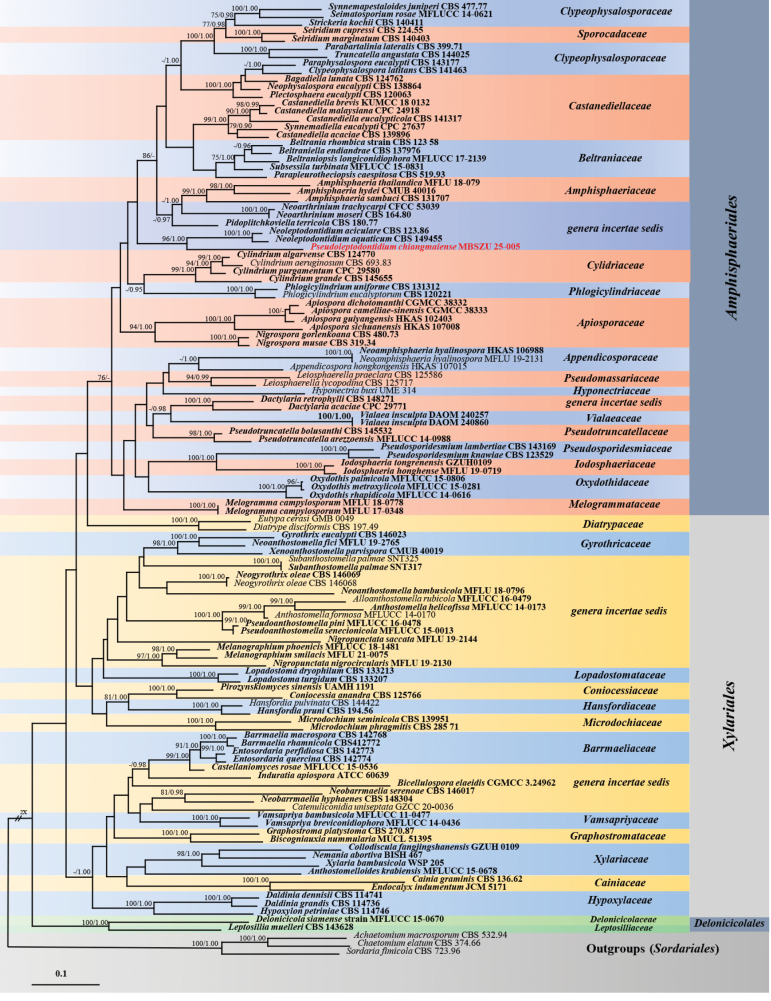
Phylogram generated from Maximum Likelihood analysis of 119 specimens belonging to the subclass Xylariomycetidae of the combined ITS, LSU, *RPB2* and *TUB* genes. *Achaetomiummacrosporum* CBS 532.94, *Chaetomiumelatum* CBS 374.66 and *Sordariafimicola* CBS 723.96 were used as the outgroup. The numbers above branches show bootstrap percentages (left) and Bayesian Posterior Probabilities (right). Bootstrap values ≥ 75% and Bayesian Posterior Probabilities ≥ 0.95 are shown. The scale bar reflects the estimated number of nucleotide substitutions per site. The fungal strains in this study are red. Type species are bold.

The phylogram of Xylariomycetidae showed that MBSZU 25-005 clustered amongst families and taxa in Amphisphaeriales. This strain also formed a sister clade to *Neoleptodontidiumaciculare* CBS 123.86 and *N.aquaticum* CBS 149455 (BS 96% and PP 1.00; Fig. 4).

### ﻿Taxonomy

#### ﻿ Penicillium terrae

Taxon classificationFungiEurotialesAspergillaceae

Thitla, Monkai, Lumyong & Hongsanan sp. nov.

09B14EF1-E0CB-59F9-911D-B9D8B1C175F1

857423

##### Etymology.

The specific epithet *terrae* refers to the soil substrate, from which this species was isolated.

##### Holotype.

Thailand • Chiang Mai Province, Mae Taeng District, Papae, on soil in the forest dump-sites, 20 June 2024, T. Thitla & J. Monkai; VR040 (SZU25-005, holotype); ex-type living culture, MBSZU 24-008, dried culture permanently preserved in a metabolically inactive state, SZU25-005.

##### Colony diam.

(in mm) 7 days, 25 °C: CREA 8–11, CYA 13–18, CYAS 7–9, CZ 11–15, DG18 12–16, MEA 15–19, MEAbl 16–19, OA 13–19, PDA 12–15 and YES 9–13. 7 days, 30 °C: CYA 10–15. 7 days, 37 °C: CYA no growth.

##### Culture characteristics.

**Figure 5.**
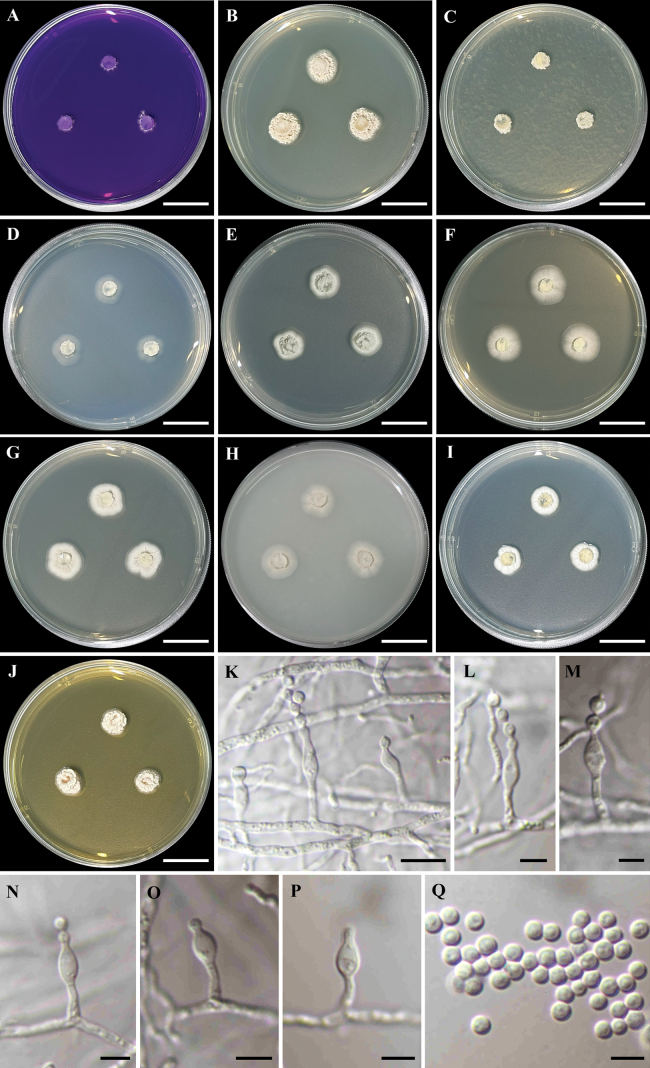
*Penicilliumterrae* (MBSZU 24-008, ex-type living culture) **A–J** colonies at 25 °C for 7 days on CREA, CYA, CYAS, CZ, DG18, MEA, MEAbl, OA, PDA and YES, respectively **K−P** conidiophores, phialides and conidia **Q** conidia. Scale bar: 2 cm (**A–J**); 10 µm (**K**); 5 µm (**L–Q**).

Colonies at 25 °C for 7 days on CREA thin colonies; acid production absent (Fig. 5A). Colonies on CYA circular, convex, wrinkled texture, entire margin; white mycelia; soluble pigment absent; reverse yellowish-brown (Fig. 5B). Colonies on CYAS barely growing, circular, raised, wrinkled texture, undulate margin; white mycelia; soluble pigment absent; reverse white (Fig. 5C). On CZ thin colonies, circular, flat, entire margin; white mycelia; soluble pigment absent; reverse white (Fig. 5D). On DG18 circular, flat, wrinkled at the centre, margin smooth and entire; grey mycelia at the centre, white mycelia at the margin; soluble pigment absent; reverse greenish-grey to light yellow (Fig. 5E). Colonies on MEA circular, flat, smooth texture, entire margin; light grey mycelia; soluble pigment absent; reverse light yellow to white (Fig. 5F). On MEAbl circular, flat, wrinkled at the centre, margin smooth and entire; light grey at the centre, white at the margin; soluble pigment absent; reverse yellowish-brown (Fig. 5G). On OA circular, flat, smooth textured, entire margin; light brown mycelia at the centre, white mycelia at the margin; soluble pigment absent; reverse white (Fig. 5H). Colonies on PDA circular, flat, wrinkled texture, entire margin; white mycelia; soluble pigment absent; reverse white to light yellow (Fig. 5I). Colonies on YES circular, convex, wrinkled texture, entire margin; white mycelia; soluble pigment absent; reverse light brown (Fig. 5J). Sporulation abundantly produces on all media.

##### Micromorphology.

Conidiophores mononematous, growing out at right angles from hyphae, unbranched, smooth, hyaline, 3–14 × 1–3 µm (Fig. 5K–P). Phialides solitary, terminal, ampulliform, smooth, hyaline, 5–12 × 1–4 µm (Fig. 5K–P). Conidia globose to subglobose, 2–4 µm diam., smooth, hyaline (Fig. 5K–N, Q). Sclerotia not observed. Sexual morph absent.

##### Additional strain examined.

Thailand • Chiang Mai Province, Mae Taeng District, Papae, on soil in the forest dump-sites, 20 June 2024, T. Thitla & J. Monkai; CMUVR039; living culture, MBSZU 24-007, dried culture permanently preserved in a metabolically inactive state, CMUVR039.

##### Habitat and distribution.

Soil; only known from Chiang Mai Province, Thailand.

##### Notes.

PenicilliumsectionExilicaulis was first established by Pitt (1980), with *P.restrictum* as the type species. This section was initially proposed to accommodate *Penicillium* species characterised by monoverticillate conidiophores and non-vesiculated stipes. Subsequently, phylogenetic studies expanded the section to include species with bi-verticillate conidiophores and those with conidiophores bearing solitary phialides (Houbraken and Samson 2011; Visagie et al. 2016a, b; da Silva et al. 2023). Species of the sect. Exilicaulis have been isolated from diverse environments, including soil, marine ecosystems, air, plants and insects (Ansari et al. 2023). Currently, this section comprises over 60 species across six series: *Alutacea*, *Citreonigra*, *Corylophila*, *Erubescentia*, *Lapidosa* and *Restricta* (Ansari et al. 2023; Visagie et al. 2024a).

*Penicilliumterrae* is classified within section Exilicaulis, series *Erubescentia*. Phylogenetically, this species is closely related to *P.laeve* and *P.ovatum* (Fig. 2). However, *P.laeve* and *P.ovatum* were unable to grow on CREA and CYAS media, while *P.terrae* can grow on these media. Regarding growth rates, *P.laeve* exhibited slower growth than *P.terrae*, including CYA (8–9 mm), DG18 (5–7 mm), OA (7–8 mm) and YES (8–9 mm) at 25 °C, as well as CYA at 30 °C (4–5 mm) (Visagie et al. 2016a). Similarly, *P.ovatum* also demonstrated slower growth compared to *P.terrae* on CYA (10–11 mm), DG18 (9–11 mm), MEA (7–8 mm) and OA (10–11 mm) at 25 °C (Visagie et al. 2016a). Micromorphologically, the phialides of *P.laeve* (4–6 µm × 2–3 µm) and *P.ovatum* (4.5–7 µm × 2–3 µm) were shorter than *P.terrae* (Visagie et al. 2016a). In terms of conidia, *P.terrae* produced globose to subglobose conidia with 2–4 µm, while *P.leave* produced globose conidia measuring 2.5–3 µm diam. and *P.ovatum* produced ellipsoidal conidia with 2–3 × 1.5–2 µm (Visagie et al. 2016a). Furthermore, a pairwise nucleotide comparison between *P.terrae* and *P.laeve* showed differences of 0.86% (5/581 bp, including gaps) in ITS, 2.87% (13/453 bp, including gaps) in *TUB*, 2.62% (13/497 bp, including gaps) in *CAM* and 1.39% (13/938 bp, including gaps) in *RPB2*. Similarly, the comparison between *P.terrae* and *P.ovatum* revealed nucleotide differences of 2.64% (15/569 bp, including gaps) in ITS, 14.41% (65/451 bp, including gaps) in *TUB*, 17.74% (91/513 bp, including gaps) in *CAM* and 12.37% (116/938 bp, including gaps) in *RPB2*.

#### ﻿ Penicillium chiangmaiense

Taxon classificationFungiEurotialesAspergillaceae

Thitla, Monkai, Lumyong & Hongsanan sp. nov.

23201173-0C3E-5F03-8787-A42BD223E573

857424

##### Etymology.

The specific epithet “*chiangmaiense*” refers to the type locality “Chiang Mai Province, Thailand”.

##### Holotype.

Thailand • Chiang Mai Province, Mae Rim District, Mae Sa, on soil in the forest dump-sites, 27 June 2024, T. Thitla & J. Monkai; VR005 (SZU25-006, holotype); ex-type living culture, MBSZU 24-009, dried culture permanently preserved in a metabolically inactive state, SZU25-006.

##### Colony diam.

(in mm) 7 days, 25 °C: CREA 40–44, CYA 50–52, CYAS 35–38, CZ 48–49, DG18 34–39, MEA 47–51, MEAbl 51–53, OA 53–54, PDA 49–50 and YES 32–38. 7 days, 30 °C: CYA 59–61. 7 days, 37 °C: CYA 55–56.

##### Culture characteristics.

**Figure 6.**
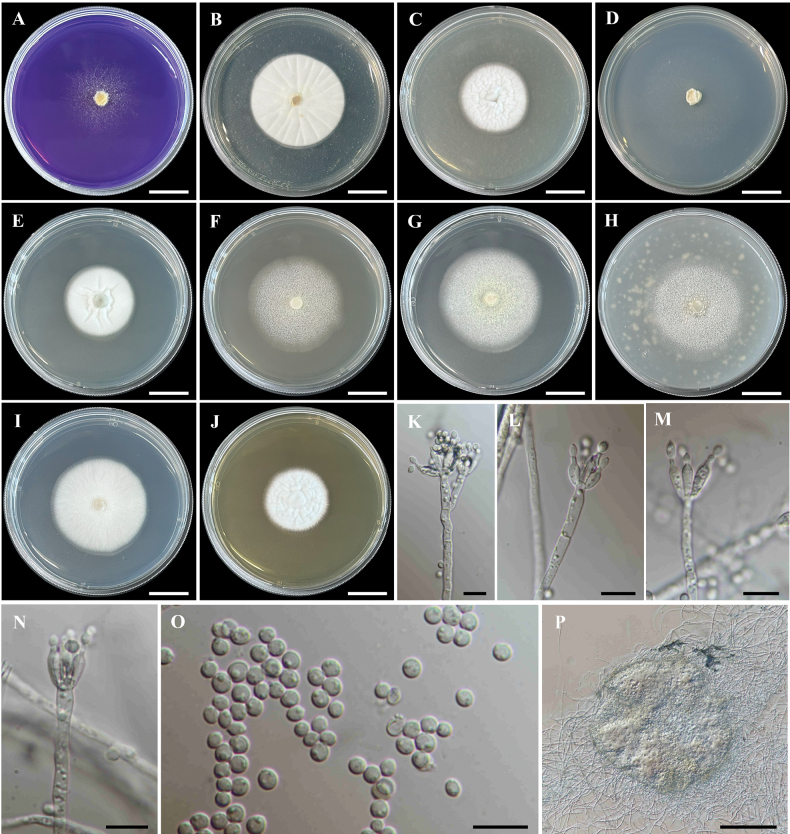
*Penicilliumchiangmaiense* (MBSZU 24-009, ex-type living culture) **A–J** colonies at 25 °C for 7 days on CREA, CYA, CYAS, CZ, DG18, MEA, MEAbl, OA, PDA and YES, respectively **K−N** conidiophores, phialides and conidia **O** conidia **P** sclerotia produced on culture media. Scale bar: 2 cm (**A–J**); 10 µm (**K–O**); 100 µm (**P**).

Colonies at 25 °C for 7 days on CREA thin colonies; acid production absent (Fig. 6A). Colonies on CYA and CYAS wrinkled texture, velvety, circular, flat, entire margin; white mycelia; soluble pigment absent; reverse light brown (Fig. 6B, C). On CZ, thin colonies, circular, flat, filamentous margin; white mycelia; soluble pigment absent; reverse white (Fig. 6D). On DG18, wrinkled texture, velvety, circular, flat, entire margin; white mycelia; soluble pigment absent; reverse white to pale yellow (Fig. 6E). Colonies on MEA and MEAbl smooth texture, circular, flat, entire margin; pale yellow at the centre, white at the margin; soluble pigment absent; reverse pale brown to white (Fig. 6F, G). On OA, smooth textured, velvety, circular, flat, entire margin; white mycelia; soluble pigment absent; reverse light yellow to white (Fig. 6H). Colonies on PDA circular, flat, smooth texture, entire margin; white mycelia; soluble pigment absent; reverse white to light yellow (Fig. 6I). Colonies on YES circular, flat, wrinkled texture, velvety, entire margin; white mycelia; soluble pigment absent; reverse brownish-yellow (Fig. 6J). Sporulation abundantly produces on DG18, MEA and MEAbl media. Sclerotia produces MEA, MEAbl and OA (Fig. 6P).

##### Micromorphology.

Conidiophores monoverticillate, sometimes divaricate. Stipes hyaline, smooth–walled, 80–270 × 2–3 µm (Fig. 6K–N). Phialides terminal, ampulliform, hyaline, smooth–walled 6–17 × 2–3.5 µm (Fig. 6K–N). Conidia globose to subglobose, 2–4 µm diam., smooth, hyaline (Fig. 6K–O). Sclerotia pale brown to brown, globose to irregular, 180–260 µm diam. (Fig. 6P). Sexual morph absent.

##### Additional strain examined.

Thailand • Chiang Mai Province, Mae Rim District, Mae Sa, on soil in the forest dump-sites, 27 June 2024, T. Thitla & J. Monkai; CMUVR005-2; living culture, MBSZU 24-010, dried culture permanently preserved in a metabolically inactive state, CMUVR005-2.

##### Habitat and distribution.

Soil; only known from Chiang Mai Province, Thailand.

##### Notes.

PenicilliumsectionLanata-Divaricata was established by Thom (1930) to include species with biverticillate conidiophores, which usually contain a main conidiophore axis and metulae that diverge (referred to as divaricate conidiophores), as well as broadly spreading colonies (Houbraken and Samson 2011; Pangging et al. 2021). Species within this section have been isolated from various sources, including soil, air, fluvial sediments and plants (Nóbrega et al. 2024). Currently, the section is divided into five series: *Dalearum*, *Janthinella*, *Oxalica*, *Rolfsiorum* and *Simplicissima* (Ansari et al. 2023; Visagie et al. 2024a).

*Penicilliumchiangmaiense* is classified within section Lanata-Divaricata, series *Janthinella*. In the phylogenetic tree (Fig. 3), the new species is closely related to *P.brefeldianum*, *P.limosum* and *P.michoacanense*. However, *P.brefeldianum* produces sexual structures on cornmeal agar and *P.limosum* produces on CZ, MEA and OA, while *P.chiangmaiense* does not exhibit any sexual features (Dodge 1933; Ueda 1995). Furthermore, the growth rate of *P.limosum* on MEA (42 mm in 14 days) was slower than that of *P.chiangmaiense* (47–51 mm in 7 days) (Ueda 1995). In the case of *P.michoacanense*, the stipes (15–60 × 1–1.5 µm) and phialides (4–5 × 1.5 µm) were shorter than those of *P.chiangmaiense* (stipes 80–270 × 2–3 µm; phialides 6–17 × 2–3.5 µm) (Rodríguez-Andrade et al. 2021). Moreover, *P.michoacanense* produced weak acid on CREA, while *P.chiangmaiense* does not produce it (Rodríguez-Andrade et al. 2021). Additionally, the pairwise nucleotide comparison of *P.chiangmaiense* with related species revealed significant differences. The comparison of *P.chiangmaiense* to *P.brefeldianum* showed 0.90% (5/556 bp) difference in ITS, 4.73% (21/444 bp) in *TUB*, 4.28% (24/561 bp) in *CAM* and 1.46% (11/755 bp) in *RPB2*, including gaps. Differences in *P.chiangmaiense* and *P.limosum* were 1.09% (6/548 bp) in ITS, 5.00% (22/440 bp) in *TUB*, 6.28% (35/557 bp) in *CAM* and 0.93% (7/755 bp) in *RPB2*, including gaps. In comparison between *P.chiangmaiense* and *P.michoacanense*, the differences were 0.73% (4/548 bp) in ITS, 2.84% (11/388 bp) in *TUB*, 8.35% (34/407 bp) in *CAM* and 1.61% (12/745 bp) in *RPB2*, including gaps.

#### ﻿ Pseudoleptodontidium

Taxon classificationFungiEurotialesAspergillaceae

Thitla, Monkai, Lumyong & Hongsanan gen. nov.

3F286329-65F9-54EA-8613-7D59A9B3CE19

857466

##### Etymology.

The name refers to its morphological similarity to *Leptodontidium*.

##### Classification.

Sordariomycetes, Xylariomycetidae, Amphisphaeriales, *incertae sedis*.

Asexual morph: Mycelium composed of hyaline to black, thin- to thick-walled, smooth, branched, septate. Conidiophores arising from hyphae, solitary, erect, cylindrical, pale brown to dark brown, thick-walled, occasionally roughened on lower part, septate. Conidiogenous cells terminal and intercalary on conidiophores, occasionally lateral on hyphae, obclavate, sympodially proliferate, denticulate, hyaline to pale brown, smooth, septate. Conidia hyaline, smooth, aseptate, subglobose to ellipsoidal, slightly curved. Chlamydospores solitary, terminal on hyphae, medium brown to dark brown, smooth, thick-walled, aseptate, subglobose. Sexual morph: absent.

##### Type species.

*Pseudoleptodontidiumchiangmaiense* Thitla, Monkai, Lumyong & Hongsanan, sp. nov.

##### Notes.

Hernández-Restrepo et al. (2017) established *Leptodontidium* in Leptodontidiaceae (Helotiales, Leotiomycetes), characterised by erect conidiophores and conidiogenous cells with a long rachis bearing denticles, as well as the presence of a *Beauveria*-like synasexual morph. *Neoleptodontidium* was introduced by Crous et al. (2023) due to its morphological resemblance to *Leptodontidium*, but it differs in having minute, terminal and lateral exophiala-like phialides. Based on LSU phylogeny, the type species of *Neoleptodontidium* (*N.aquaticum*) clustered with *Leptodontidiumaciculare* (Crous et al. 2023). Hence, Crous et al. (2023) transferred *L.aciculare* to *Neoleptodontidium* as *N.aciculare* by the morphological and phylogenetic congruence.

*Pseudoleptodontidium* is morphologically similar to *Neoleptodontidium*, sharing septate, subcylindrical conidiophores, terminal and lateral phialidic conidiogenous cells and aseptate subcylindrical conidia (Crous et al. 2023). However, *Pseudoleptodontidium* can be distinguished from *Neoleptodontidium* by its obclavate, sympodially proliferating, denticulate conidiogenous cells and subglobose to ellipsoidal conidia. The phylogeny, based on a combined ITS, LSU, *RPB2* and *TUB* dataset, revealed that *Pseudoleptodontidium* forms an independent lineage, sister to *Neoleptodontidium* with significant support (BS 96% ML and PP 1.00; Fig. 4). Although Crous et al. (2023) placed *Neoleptodontidium* in Xylariales genera *incertae sedis*, our phylogeny indicates that *Pseudoleptodontidium* and *Neoleptodontidium* are closely related to the Amphisphaeriaceae, Cylidriaceae, Phlogicylindriaceae and Amphisphaeriales genera *incertae sedis* (*Neoarthrinium*, *Pidoplitchkoviella*) (Fig. 4). Therefore, due to their distinct morphology and phylogeny, *Pseudoleptodontidium* is introduced as a genus *incertae sedis* in Amphisphaeriales, with *Ps.chiangmaiense* designated as the type species.

#### ﻿ Pseudoleptodontidium chiangmaiense

Taxon classificationFungiEurotialesAspergillaceae

Thitla, Monkai, Lumyong & Hongsanan sp. nov.

12A6C18E-1B92-5BE2-9E00-013554B2933C

857467

##### Etymology.

The specific epithet *chiangmaiense* refers to the type locality, Chiang Mai Province, Thailand.

##### Holotype.

Thailand•Chiang Mai Province, Mueang Chiang Mai District, Su Thep, on soil in the forest dump-sites, 21 June 2024, T. Thitla & J. Monkai; VR044 (SZU25-007, holotype); ex-type living culture, MBSZU 25-005, dried culture permanently preserved in a metabolically inactive state, SZU25-007.

##### Colony diam.

(in mm) 14 days, 25 °C: PDA 36–40 and MEA 31–38.

##### Culture characteristics.

**Figure 7.**
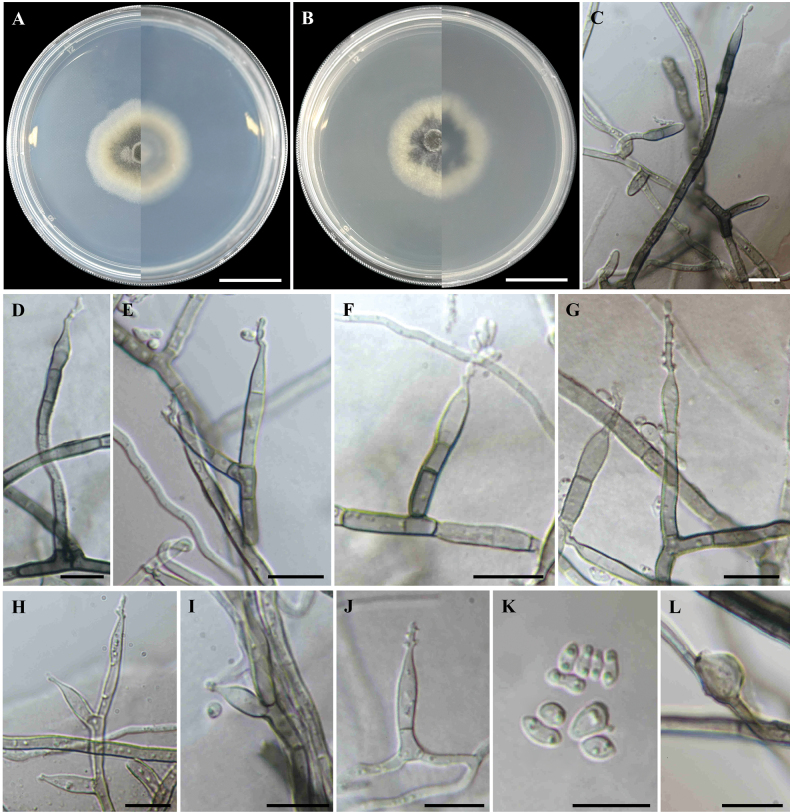
*Pseudoleptodontidiumchiangmaiense* (MBSZU 25-005, ex-type living culture) **A, B** colonies from surface and reverse view at 25 °C for 14 days on PDA and MEA, respectively **C−J** conidiophores, conidiogenous cells and conidia **K** conidia **L** chlamydospore. Scale bar: 2 cm (**A, B**); 10 µm (**C–L**).

Colonies at 25 °C for 14 days on PDA velvety, circular, flat, entire margin; dark green at the centre, greenish-yellow at the middle, white at the margin; soluble pigment absent; reverse dark green to pale yellow, white at the margin (Fig. 7A). Colonies on MEA velvety, circular, flat, entire margin; dark green to black at the centre, yellowish-green to white at the margin; soluble pigment absent; reverse dark green at the centre, pale yellow to white at the margin (Fig. 7B).

##### Micromorphology.

Mycelium composed of hyaline to black, thin- to thick-walled, smooth, branched, septate, 2–4.5 µm diam. hyphae (Fig. 7C–L). Conidiophores arising from hyphae, solitary, erect, cylindrical, pale brown to dark brown, thick-walled, occasionally roughened on lower part, septate, 7–70 × 2.5–5 µm (Fig. 7C–G). Conidiogenous cells terminal and intercalary on conidiophores, occasionally lateral on hyphae, obclavate, sympodially proliferate, denticulate, hyaline to pale brown, smooth, 0-1 septate, 7.5–26 × 3–5 µm (Fig. 7C–J). Conidia hyaline, smooth, aseptate, subglobose to ellipsoidal, slightly curved, 3–7.5 × 1.5–4 µm (Fig. 7K). Chlamydospores solitary, terminal on hyphae, medium brown to dark brown, smooth, thick-walled, aseptate, subglobose, 6–8 × 4.5–6 µm (Fig. 7L). Sexual morph absent.

##### Habitat and distribution.

Soil; only known from Chiang Mai Province, Thailand.

##### Notes.

*Pseudoleptodontidiumchiangmaiense* has a close relationship with *Neoleptodontidiumaciculare* and *N.aquaticum* (Fig. 4). However, their morphological characteristics are distinct: *Ps.chiangmaiense* has broader conidiogenous cells (7.5–26 × 3–5 µm) than *N.aciculare* (15–30 × 2–3 µm) and *N.aquaticum* (10–30 × 2–2.5 µm) and larger conidia (3–7.5 × 1.5–4 µm) than *N.aciculare* (3–4 × 1–2 µm) and *N.aquaticum* (3–4 × 1.5 µm) (Rao and De Hoog 1986; Hernández-Restrepo et al. 2017). The pairwise nucleotide comparison between *Ps.chiangmaiense* and *N.aciculare* revealed differences of 16.38% (95/580 bp, including gaps) in the ITS region and 4.92% (40/813 bp, including gaps) in the LSU region. Additionally, the comparison between *Ps.chiangmaiense* and *N.aquaticum* revealed differences of 16.23% (87/536 bp, including gaps) in the ITS region and 4.80% (39/813 bp, including gaps) in the LSU region.

## ﻿Discussion

This study identifies a new genus in Xylariomycetidae, namely *Pseudoleptodontidium*, accommodating *Ps.chiangmaiense* sp. nov., along with two new species of *Penicillium*: *P.terrae* in section Exilicaulis and *P.chiangmaiense* in section Lanata-Divaricata. These species were isolated from soil collected in forest dump-sites in Chiang Mai Province, Thailand. They were characterised through morphological observations and multigene phylogenetic analyses (Figs 2–6).

*Penicillium* is a highly impactful genus, with species ranging from mycotoxin-producing plant pathogens and opportunistic animal and human pathogens to valuable sources of enzymes, antibiotics and bioactive compounds (Oshikata et al. 2013; Perrone and Susca 2017; Costa et al. 2019; Toghueo and Boyom 2020; Wolski 2023; Suwannarach et al. 2024). The genus was proposed by Link (1809) and currently comprises two subgenera, 34 sections, 102 series and 535 accepted species (Visagie et al. 2024a). *Penicillium* was traditionally identified, based on macro-morphology (such as colony characteristics and pigment production) and micro-morphology (including conidiophores, branches, metula, phialides and conidia) (Khuna et al. 2023). However, relying solely on morphological characteristics has proven insufficient for accurate identification. Consequently, an integrated approach combining morphology, molecular data and extrolite analysis is currently used to identify species within the genus *Penicillium* (Visagie et al. 2014; Labuda et al. 2021; Nguyen and Pham 2022; Visagie et al. 2024a, 2024b). In section Exilicaulis, key genetic data for species identification include the internal transcribed spacer region (ITS), beta-tubulin (*TUB*), calmodulin (*CAM*) and RNA polymerase II subunit (*RPB2*) genes (Visagie et al. 2016c). Initially, *P.laeve* and *P.ovatum* were introduced under the genus *Torulomyces* as *T.laevis* and *T.ovatus*, respectively (Ando et al. 1998). Subsequently, phylogenetic analyses using RNA polymerase II largest subunit (*RPB1*), *RPB2*, the protein required for processing of 20S pre-rRNA in the cytoplasm (*Tsr1*) and the subunit of the cytosolic chaperonin Cct ring complex (*Cct8*) led to transfer to PenicilliumsectionTorulomyces (Houbraken and Samson 2011). Visagie et al. (2016a) reclassified these species into section Exilicaulis using ITS, *TUB*, *CAM* and *RPB2* sequence data. Currently, *P.laeve* and *P.ovatum* belong to the series *Erubescentia*, characterised by species with monoverticillate conidiophores, short stipes and the ability to grow at 37 °C (Houbraken et al. 2020). However, both *P.laeve* and *P.ovatum*, along with *P.terrae*, produce conidiophores with solitary phialides and were unable to grow at 37 °C. Additionally, the phylogenetic clade of these species formed a basal clade with other species in this series with strong support (BS 97% and PP 1.00) (Fig. 2). In our opinion, this distinct clade may represent a potential new series within section Exilicaulis and should be further studied in the future.

Prior to this study, *P.laeve* was the only species in section Exilicaulis reported from Thailand (Ando et al. 1998). The discovery of *P.terrae* from soil in Thailand marks the second species from this section identified in the country. Furthermore, this new species represents the 69^th^ global species in section Exilicaulis, as shown in Suppl. material 1: table S1, excluding *P.janthinellum* and *P.limosum*. In addition, this study proposed a new species, *P.chiangmaiense* in section Lanata-Divaricata, which is the second species recorded in Thailand from this section, following the first species (*P.singorense*) described by Visagie et al. (2014b). Additionally, this new species represents the 108^th^ global species in section Lanata-Divaricata, as outlined in Suppl. material 1: table S2, excluding *P.alogum* and *P.stolkiae*.

Ecologically, *Penicillium* species have been isolated from different environments (Ansari et al. 2023; Nóbrega et al. 2024). For instance, *P.chiangmaiense* and its closely-related species, including *P.brefeldianum*, *P.limosum* and *P.michoacanense*, have been found in the human digestive tract, marine sediments and soil (Dodge 1933; Ueda 1995; Rodríguez-Andrade et al. 2021). Similarly, *P.terrae* and its relatives, including *P.laeve* and *P.ovatum*, have primarily been reported from soil, with *P.laeve* specifically found in forest soils in Thailand (Ando et al. 1998). These findings highlight the ecological plasticity of *Penicillium* species, which can potentially thrive in disturbed ecosystems. Future studies examining their functional traits and metabolic profiles could further enhance better understanding of their ecological significance.

Xylariomycetidae is a large subclass within Sordariomycetes comprising numerous taxa that are polyphyletic and paraphyletic (Wendt et al. 2017; Daranagama et al. 2018; Konta et al. 2020; Samarakoon et al. 2022). The taxonomic classification of Xylariomycetidae has undergone considerable change (Maharachchikumbura et al. 2016; Samarakoon et al. 2016, 2022). Earlier, Amphisphaeriales was considered a synonym of Xylariales (Maharachchikumbura et al. 2016). However, based on morphology, molecular data, divergence estimates and ancestral state reconstruction, Samarakoon et al. (2016, 2022) subsequently reclassified Amphisphaeriales, Delonicicolales and Xylariales in Xylariomycetidae. Molecular phylogeny, based on concatenated ITS, LSU, *RPB2*, *TUB* and *TEF-1α* sequence data, demonstrated the placement of Amphisphaeriales in a sister clade to Xylariales (Samarakoon et al. 2022), which is consistent with our study (Fig. 4). However, we did not incorporate *TEF-1a* into the phylogenetic tree, as the number of taxa with available sequence data was low. The classification of taxa within Xylariomycetidae remains ambiguous, as more than 50 *incertae sedis* genera await taxonomic resolution (Samarakoon et al. 2022). Likewise, our study was unable to assign the novel genus *Pseudoleptodontidium* to any family within the Xylariomycetidae (Fig. 4). The new lineage of *Pseudoleptodontidium* and *Neoleptodontidium* also lacks significant statistical support for placement within other taxa and families in Amphisphaeriales, though it is likely linked to Amphisphaeriaceae, Cylidriaceae and Phlogicylindriaceae (Fig. 4). Further taxonomic and phylogenetic studies, including the collection of new specimens and the examination of additional isolates, are necessary to confirm the familial placement of *Pseudoleptodontidium* and *Neoleptodontidium*.

Members of Xylariomycetidae have a worldwide distribution and occupy various ecological niches, including saprobes, endophytes and pathogens (U’Ren et al. 2016; Daranagama et al. 2018; Sugita et al. 2022; Samarakoon 2024). Recently, several new taxa have been reported as saprobes on dead plant materials from Thailand (Monkai et al. 2022; Afshari et al. 2023; Samarakoon et al. 2023; Karimi et al. 2023, 2024; Samarakoon 2024; Thakshila et al. 2024). In this study, *Pseudoleptodontidium* was isolated from soil associated with a forest dump-site in Thailand, whereas *Neoleptodontidium* species have been found in hydroponic water and decomposing wood in the USA and India (Rao and De Hoog 1986; Hernández-Restrepo et al. 2017). This demonstrates that these taxa have a broad distribution range, highlighting their adaptability in diverse environments.

These findings significantly contribute to our understanding of fungal diversity and ecology, particularly within the Ascomycota and highlight the richness and diversity of soil fungal communities in Thailand. *Penicillium* and some Xylariomycetidae taxa, such as *Amphisphaeria*, *Annulohypoxylon* and *Hypoxylon* are recognised for possessing a wide variety of secondary metabolites, which have prospective agricultural and therapeutic uses (Toghueo and Boyom 2020; Becker and Stadler 2021; Wang et al. 2023a; Wolski 2023). The discovery of novel fungi in forest dump areas presents an opportunity to explore and characterise these fungi for various applications. Therefore, further research is necessary to evaluate the capabilities of new fungal strains for extracellular enzyme production and the degradation of synthetic materials.

## Supplementary Material

XML Treatment for Penicillium terrae

XML Treatment for Penicillium chiangmaiense

XML Treatment for Pseudoleptodontidium

XML Treatment for Pseudoleptodontidium chiangmaiense

## ﻿Additional information

### Author ORCIDs

Tanapol Thitla https://orcid.org/0000-0003-3092-0679

Jutamart Monkai https://orcid.org/0000-0001-6043-0625

Weiqian Meng https://orcid.org/0009-0006-3840-0992

Surapong Khuna https://orcid.org/0000-0003-1999-4001

Ning Xie https://orcid.org/0000-0002-5866-8535

Sinang Hongsanan https://orcid.org/0000-0003-0550-3152

Saisamorn Lumyong https://orcid.org/0000-0002-6485-414X

### Data availability

The datasets generated during and/or analysed during the current study are available in the MycoBank repository (included in the manuscript) and GenBank (included in Suppl. material 1: tables S1–S3). Additionally, the datasets generated during and/or analysed during the current study are available from the corresponding author on reasonable request.

## Supplementary materials

Supplementary material 1 Additional information Data type docx Explanation note **table S1.** GenBank accession numbers of PenicilliumsectionExilicaulis used in multi-locus phylogenetic analysis. **table S2.** GenBank accession numbers of PenicilliumsectionLanata-Divaricata used in multi-locus phylogenetic analysis. **table S3.** GenBank accession numbers of taxa in Xylariomycetidae used in multi-genes phylogenetic analysis. https://binary.pensoft.net/file/1318660This dataset is made available under the Open Database License (http://opendatacommons.org/licenses/odbl/1.0/). The Open Database License (ODbL) is a license agreement intended to allow users to freely share, modify, and use this Dataset while maintaining this same freedom for others, provided that the original source and author(s) are credited.Tanapol Thitla, Jutamart Monkai, Weiqian Meng, Surapong Khuna, Ning Xie, Sinang Hongsanan, Saisamorn Lumyong
