# Supplementary material for: ﻿Two new species of Penicillium and a new genus in Xylariomycetidae from the forest dump-sites in Chiang Mai, Thailand
Source: MycoKeys. 2025 Apr 29;116:275–301. doi: 10.3897/mycokeys.116.150635 (PMC12056519; doi:10.3897/mycokeys.116.150635)
Supplement: Supplementary material 1 — Additional information [file mycokeys-116-275-s001.docx]

**Table S1** GenBank accession numbers of *Penicillium* section *Exilicaulis* used in multi-locus phylogenetic analysis.

| **Species** | **Strain numbers** | ***ITS*** | ***TUB*** | ***CAM*** | ***RPB2*** |
| --- | --- | --- | --- | --- | --- |
| *P*. *allaniae* | BRIP 74886a ^T^ | OP903476 | OP921959 | OP921957 | OP921958 |
| *P*. *alutaceum* | NRRL 5812 ^T^ | AF033454 | KJ834430 | KP016768 | JN121489 |
| *P*. *amapaense* | INPA-AP07 ^T^ | OL764382 | OL782590 | OL782584 | ON854925 |
| *P*. *aotearoae* | CBS 140999 ^T^ | KT887874 | KT887835 | KT887796 | MN969174 |
| *P*. *arabicum* | CBS 414.69 ^T^ | KC411758 | KP016750 | KP016770 | KP064574 |
| *P*. *archerae* | BRIP 72549c ^T^ | OP903477 | OP921961 | – | OP921960 |
| *P*. *atrolazulinum* | CBS 139136 ^T^ | JX140913 | JX141077 | JX157416 | KP064575 |
| *P*. *atrosanguineum* | CBS 380.75 ^T^ | JN617706 | KJ834435 | KP016771 | JN406557 |
| *P*. *burgense* | CBS 325.89 ^T^ | KC411736 | KJ834437 | KP016772 | JN406572 |
| *P*. *canis* | NRRL 62798 ^T^ | KJ511291 | KF900167 | KF900177 | KF900196 |
| *P*. *catenatum* | CBS 352.67 ^T^ | KC411754 | KJ834438 | KP016774 | JN121504 |
| *P*. *chalabudae* | CBS 219.66 ^T^ | KP016811 | KP016748 | KP016767 | KP064572 |
| *P*. *cinerascens* | NRRL 748 ^T^ | AF033455 | JX141041 | JX157405 | KP064614 |
| *P*. *cinereoatrum* | CBS 222.66 ^T^ | KC411700 | KJ834442 | KP125335 | JN406608 |
| *P*. *citreonigrum* | NRRL 761 ^T^ | AF033456 | EF198621 | EF198628 | JN121474 |
| *P*. *citreosulfuratum* | DTO 290-I4 ^T^ | KP016814 | KP016753 | KP016777 | KP064615 |
| *P*. *consobrinum* | CBS 139144 ^T^ | JX140888 | JX141135 | JX157453 | KP064619 |
| *P*. *corylophilum* | NRRL 802 ^T^ | AF033450 | JX141042 | KP016780 | KP064631 |
| *P*. *cravenianum* | CBS 139138 ^T^ | JX140900 | JX141076 | JX157418 | KP064636 |
| *P*. *danzhouense* | CGMCC 3.25204 ^T^ | ON563150 | ON231295 | ON470838 | – |
| *P*. *decumbens* | CBS 230.81 ^T^ | AY157490 | KJ834446 | KP016782 | JN406601 |
| *P*. *diabolicalicense* | CBS 140967 ^T^ | KT887840 | KT887801 | KT887762 | MN969175 |
| *P*. *dimorphosporum* | NRRL 5207 ^T^ | AF081804 | KJ834448 | KP016783 | JN121517 |
| *P*. *dravuni* | F01V25 ^T^ | AY494856 | – | – | – |
| *P*. *erubescens* | NRRL 6223 ^T^ | AF033464 | HQ646566 | EU427281 | JN121490 |
| *P*. *fagi* | CBS 689.77 ^T^ | MH861113 | KJ834449 | KP016784 | JN406540 |
| *P*. *flemingii* | CGMCC 3.25158 ^T^ | OQ867293 | OR051093 | OR051270 | OR051441 |
| *P*. *fundyense* | DAOMC 250519 ^T^ | KT887853 | KT887814 | KT887775 | MN969176 |
| *P*. *guttulosum* | NRRL 907 ^T^ | HQ646592 | HQ646576 | HQ646587 | KP064639 |
| *P*. *hemitrachum* | CBS 139134 ^T^ | FJ231003 | JX141048 | JX157526 | KP064642 |
| *P*. *hermansii* | DTO 079-D5 ^T^ | MG333472 | MG386214 | MG386229 | MG386242 |
| *P*. *heteromorphum* | CBS 226.89 ^T^ | KC411702 | KJ834455 | KP016786 | JN406605 |
| *P*. *janthinellum* ^OUT^ | CBS 340.48 ^T^ | GU981585 | GU981625 | MN969268 | JN121497 |
| *P*. *katangense* | NRRL 5182 ^T^ | AF033458 | KP016757 | KP016788 | KP064646 |
| *P*. *krskae* | CBS 147776 ^T^ | MW794123 | MW774594 | MW774595 | MW774593 |
| *P*. *kurssanovii* | NRRL 3381 ^T^ | EF422849 | KP016758 | KP016789 | KP064647 |
| *P*. *labradorum* | DI19-20 ^T^ | MK881918 | MK887898 | MK887899 | MK887900 |
| *P*. *laeve* | DTO 270G8 ^T^ | KF667369 | KF667365 | KF667367 | KF667371 |
| *P*. *lapidosum* | CBS 343.48 ^T^ | MH856379 | KJ834465 | FJ530984 | JN121500 |
| *P*. *limosum* ^OUT^ | CBS 339.97 ^T^ | GU981568 | GU981621 | MN969271 | KF296433 |
| *P*. *maclennaniae* | CBS 198.81 ^T^ | KC411689 | KJ834468 | KP016791 | KP064648 |
| *P*. *melinii* | CBS 218.30 ^T^ | MH855124 | KJ834471 | KP016792 | JN406613 |
| *P*. *menonorum* | NRRL 50410 ^T^ | HQ646591 | HQ646573 | HQ646584 | KF900194 |
| *P*. *meridianum* | NRRL 5814 ^T^ | AF033451 | KJ834472 | KP016794 | JN406576 |
| *P*. *momoii* | CBS 139157 ^T^ | JX140895 | JX141073 | JX157479 | KP064673 |
| *P*. *namyslowskii* | NRRL 1070 ^T^ | AF033463 | JX141067 | KP016795 | JF417430 |
| *P*. *nepalense* | CBS 203.84 ^T^ | KC411692 | KJ834474 | KP016796 | JN121453 |
| *P*. *ovatum* | DTO270G7 ^T^ | KF667370 | KF667366 | KF667368 | KF667372 |
| *P*. *pagulum* | CBS 139166 ^T^ | JX140898 | JX141070 | JX157519 | KP064655 |
| *P*. *parvofructum* | CBS 141690 ^T^ | LT559091 | LT627645 | LT627646 | MN969197 |
| *P*. *parvum* | NRRL 2095 ^T^ | AF033460 | HQ646568 | KF900173 | JN406559 |
| *P*. *philippinense* | CBS 623.72 ^T^ | KC411770 | KJ834482 | KP016799 | JN406543 |
| *P*. *pimiteouiense* | NRRL 25542 ^T^ | AF037431 | HQ646569 | HQ646580 | JN406650 |
| *P*. *punicae* | CNUFC-FP 2-1 ^T^ | – | KX839673 | KX839671 | KX839675 |
| *P*. *raciborskii* | NRRL 2150 ^T^ | AF033447 | JX141069 | KP016800 | JN406607 |
| *P*. *repensicola* | CBS 139160 ^T^ | JX140893 | JX141150 | JX157490 | KP064660 |
| *P*. *restrictum* | NRRL 1748 ^T^ | AF033457 | KJ834486 | KP016803 | JN121506 |
| *P*. *rhizophilum* | CBS 149737 ^T^ | OP824727 | OP846100 | OP846104 | OP846107 |
| *P*. *rubefaciens* | CBS 145.83 ^T^ | MH861557 | KJ834487 | KP016804 | JN406627 |
| *P*. *rubidurum* | NRRL 6033 ^T^ | AF033462 | HQ646574 | HQ646585 | JN406545 |
| *P*. *silybi* | CBS 147777 ^T^ | KF367458 | MW774592 | MW774591 | AB860248 |
| *P*. *smithii* | CBS 276.83 ^T^ | KC411723 | KJ834492 | KP016806 | JN406589 |
| *P*. *striatisporum* | NRRL 26877 ^T^ | AF038938 | JX141156 | KP016807 | JN406538 |
| *P*. *subturcoseum* | CV110 ^T^ | FJ231006 | JX141161 | JX157532 | KP064674 |
| *P*. *tenue* | CGMCC 3.25205 ^T^ | ON563151 | ON231296 | ON470839 | ON470842 |
| ***P*. *terrae*** | **MBSZU 24-007** | **PV036868** | **PV054938** | **PV054942** | **PV036875** |
| ***P*. *terrae*** | **MBSZU 24-008 ^T^** | **PV036869** | **PV054939** | **PV054943** | **PV036876** |
| *P*. *terrenum* | CBS 313.67 ^T^ | MH858978 | KJ834496 | KP016808 | JN406577 |
| *P*. *vallebormidaense* | CBS 147064 ^T^ | MT316359 | MW115862 | MW115863 | MW115864 |
| *P*. *velutinum* | CBS 250.32 ^T^ | MH855309 | JX141170 | KP016810 | KP064682 |
| *P*. *vinaceum* | NRRL 739 ^T^ | AF033461 | HQ646575 | HQ646586 | JN406555 |
| *P*. *xanthomelinii* | CBS 139163 ^T^ | JX140921 | JX141120 | JX157495 | KP064683 |

Fungal species obtained in this study are bold. Superscript “T” represents ex-type species. The superscript “OUT” represents the outgroup taxa. “–” represents the absence of sequence data in the GenBank database. Acronyms of culture collections: BRIP - the culture collection held in the Queensland Plant Pathology Herbarium, Queensland, Australia; CBS - the Westerdijk Fungal Biodiversity Institute, Utrecht, the Netherlands; CGMCC - the China General Microbiological Culture Collection Center, Beijing, China; CNUFC - the Chonnam National University Fungal Collection, Gwangju, South Korea; DAOMC - the Canadian Collection of Fungal Cultures, Ontario, Canada; DTO - the Applied and Industrial Mycology group, the Westerdijk Fungal Biodiversity Institute, Utrecht, the Netherlands; INPA - the National Institute for Amazonian Research, Manaus, Brasil; MBSZU - the Culture Collection of Microbial Shenzhen University, Shenzhen, China; NRRL - the Agricultural Research Service Culture Collection, U.S. Department of Agriculture, DC, USA.

**Table S2** GenBank accession numbers of *Penicillium* section *Lanata-Divaricata* used in multi-locus phylogenetic analysis.

| **Species** | **Strain numbers** | **ITS** | ***TUB*** | ***CAM*** | ***RPB2*** |
| --- | --- | --- | --- | --- | --- |
| *P*. *abidjanum* | CBS 246.67 ^T^ | GU981582 | GU981650 | MN969234 | JN121469 |
| *P*. *alagoense* | URM 8086 ^T^ | MK804503 | MK802333 | MK802336 | MK802338 |
| *P*. *alogum* ^OUT^ | CBS 140996 ^T^ | KT887869 | KT887830 | KT887791 | MN969172 |
| *P*. *amazonense* | COAD3476 ^T^ | MT140343 | MW820833 | MW820831 | OK087392 |
| *P*. *amphipolaria* | CBS 140997 ^T^ | KT887872 | KT887833 | KT887794 | MN969177 |
| *P*. *annulatum* | CBS 135126 ^T^ | JX091426 | JX091514 | JX141545 | KF296410 |
| *P*. *araracuaraense* | CBS 113149 ^T^ | GU981597 | GU981642 | MN969237 | KF296414 |
| *P*. *ashbyae* | MST-F 22310 ^T^ | OR731310 | OR737776 | OR737766 | OR737771 |
| *P*. *ausonanum* | FMR 16948 ^T^ | LR655808 | LR655809 | LR655810 | LR655811 |
| *P*. *austrosinense* | CGMCC 3.18797 ^T^ | KY495007 | KY495116 | MN969328 | KY495061 |
| *P*. *bissettii* | CBS 140972 ^T^ | KT887845 | KT887806 | KT887767 | MN969178 |
| *P*. *brasilianum* | CBS 253.55 ^T^ | GU981577 | GU981629 | MN969239 | KF296420 |
| *P*. *brefeldianum* | CBS 235.81 ^T^ | AF033435 | GU981623 | EU021683 | KF296421 |
| *P*. *camponotum* | CBS 140982 ^T^ | KT887855 | KT887816 | KT887777 | MN969179 |
| *P*. *caperatum* | CBS 443.75 ^T^ | KC411761 | GU981660 | MN969242 | KF296422 |
| *P*. *cataractum* | CBS 140974 ^T^ | KT887847 | KT887808 | KT887769 | MN969180 |
| ***P*. *chiangmaiense*** | **MBSZU 24-009 ^T^** | **PV036870** | **PV054940** | **PV054944** | **PV036877** |
| ***P*. *chiangmaiense*** | **MBSZU 24-010** | **PV036871** | **PV054941** | **PV054945** | **PV036878** |
| *P*. *coeruleum* | CBS 141.45 ^T^ | GU981606 | GU981655 | MN969247 | KF296425 |
| *P*. *coffeatum* | CGMCC 3.25152 ^T^ | OQ870815 | OR051121 | OR051298 | OR051466 |
| *P*. *cremeogriseum* | CBS 223.66 ^T^ | GU981586 | GU981624 | MN969250 | KF296426 |
| *P*. *curticaule* | CBS 135127 ^T^ | FJ231021 | JX091526 | JX141536 | KF296417 |
| *P*. *daleae* | CBS 211.28 ^T^ | GU981583 | GU981649 | MN969251 | KF296427 |
| *P*. *diatomitis* | CCF 3904 ^T^ | FJ430748 | HE651133 | LT970912 | LT797560 |
| *P*. *donggangicum* | CGMCC 3.15900 ^T^ | MW946996 | MZ004914 | MZ004918 | MW979253 |
| *P*. *echinulonalgiovense* | CBS 328.59 ^T^ | GU981587 | GU981631 | KX961269 | KX961301 |
| *P*. *ehrlichii* | CBS 324.48 ^T^ | GU981578 | GU981652 | MN969253 | KF296428 |
| *P*. *elleniae* | CBS 118135 ^T^ | GU981612 | GU981663 | MN969254 | KF296429 |
| *P*. *excelsum* | IBT 31516 ^T^ | KR815341 | KP691061 | KR815342 | MN969166 |
| *P*. *fengjieense* | CGMCC 3.25157 ^T^ | OQ870765 | OR051156 | OR051333 | OR051489 |
| *P*. *flaviroseum* | CGMCC 3.18805 ^T^ | KY495032 | KY495141 | MN969329 | KY495083 |
| *P*. *fructuariae-cellae* | CBS 145110 ^T^ | MK039434 | KU554679 | MK045337 | – |
| *P*. *glaucoroseum* | CBS 138908 ^T^ | MN431390 | MN969383 | MN969257 | MN969119 |
| *P*. *globosum* | CGMCC 3.18800 ^T^ | KY495014 | KY495123 | MN969330 | KY495067 |
| *P*. *griseoflavum* | CGMCC 3.18799 ^T^ | KY495011 | KY495120 | MN969331 | KY495064 |
| *P*. *griseopurpureum* | CBS 406.65 ^T^ | KF296408 | KF296467 | MN969261 | KF296431 |
| *P*. *guaibinense* | CCDCA 11512 ^T^ | MH674389 | MH674391 | MH674393 | – |
| *P*. *guangxiense* | CGMCC 3.18793 ^T^ | KY494986 | KY495095 | MN969332 | KY495045 |
| *P*. *hainanense* | CGMCC 3.18798 ^T^ | KY495009 | KY495118 | MN969333 | KY495062 |
| *P*. *hepuense* | AS 3.16039 ^T^ | MW946994 | MZ004912 | MZ004916 | MW979254 |
| *P*. *heveae* | COAD3467 ^T^ | MG751217 | MW928622 | MW928617 | OK087394 |
| *P*. *infrabuccalum* | CBS 140983 ^T^ | KT887856 | KT887817 | KT887778 | MN969181 |
| *P*. *janthinellum* | CBS 340.48 ^T^ | GU981585 | GU981625 | MN969268 | JN121497 |
| *P*. *javanicum* | CBS 341.48 ^T^ | GU981613 | GU981657 | MN969269 | JN121498 |
| *P*. *jianfenglingense* | CGMCC 3.18802 ^T^ | KY495016 | KY495125 | MN969334 | KY495069 |
| *P*. *jiaozhouwanicum* | AS 3.16038 ^T^ | MW946993 | MZ004911 | MZ004915 | MW979252 |
| *P*. *jinyunshanicum* | CGMCC 3.25162 ^T^ | OQ870766 | OR051157 | OR051334 | OR051490 |
| *P*. *koreense* | CBS 141338 ^T^ | KJ801939 | KM000846 | MN969317 | MN969159 |
| *P*. *laevigatum* | CGMCC 3.18801 ^T^ | KY495015 | KY495124 | MN969335 | KY495068 |
| *P*. *levitum* | CBS 345.48 ^T^ | GU981607 | GU981654 | MN969270 | KF296432 |
| *P*. *limosum* | CBS 339.97 ^T^ | GU981568 | GU981621 | MN969271 | KF296433 |
| *P*. *limpopoense* | CMV 012F9 ^T^ | MK450722 | MK451261 | MK451651 | MK450854 |
| *P*. *lineolatum* | CBS 188.77 ^T^ | GU981579 | GU981620 | MN969272 | KF296434 |
| *P*. *ludwigii* | CBS 417.68 ^T^ | KF296409 | KF296468 | MN969273 | KF296435 |
| *P*. *malacosphaerulum* | CBS 135120 ^T^ | FJ231026 | JX091524 | JX141542 | KF296438 |
| *P*. *mariae-crucis* | CBS 271.83 ^T^ | GU981593 | GU981630 | MN969275 | KF296439 |
| *P*. *marykayhuntiae* | BRIP 74934a ^T^ | OR271913 | OR269446 | – | OR269440 |
| *P*. *melanosporum* | FMR 17424 ^T^ | LR655192 | LR655196 | LR655200 | LR655204 |
| *P*. *meloforme* | CBS 445.74 ^T^ | KC411762 | GU981656 | MN969276 | KF296440 |
| *P*. *michoacanense* | FMR 17612 ^T^ | LR655194 | LR655198 | LR655202 | LR655206 |
| *P*. *newtonturnerae* | BRIP 74909a ^T^ | OP903478 | OP921964 | OP921962 | OP921963 |
| *P*. *nordestinense* | URM 8423 ^T^ | OV265270 | OV265324 | OV265272 | OM927721 |
| *P*. *ochrochloron* | CBS 357.48 ^T^ | GU981604 | GU981672 | MN969280 | KF296445 |
| *P*. *onobense* | CBS 174.81 ^T^ | GU981575 | GU981627 | MN969281 | KF296447 |
| *P*. *ortum* | CBS 135669 ^T^ | JX091427 | JX091520 | JX141551 | KF296443 |
| *P*. *oxalicum* | CBS 219.30 ^T^ | AF033438 | KF296462 | MN969283 | JN121456 |
| *P*. *panissanguineum* | CBS 140989 ^T^ | KT887862 | KT887823 | KT887784 | MN969182 |
| *P*. *paraherquei* | CBS 338.59 ^T^ | AF178511 | KF296465 | MN969285 | KF296449 |
| *P*. *pauciramulum* | CGMCC 3.25164 ^T^ | OQ870726 | OR051111 | OR051288 | OR051457 |
| *P*. *pedernalense* | CBS 140770 ^T^ | KU255398 | KU255396 | MN969322 | MN969184 |
| *P*. *penarojense* | CBS 113178 ^T^ | GU981570 | GU981646 | MN969287 | KF296450 |
| *P*. *piscarium* | CBS 362.48 ^T^ | GU981600 | GU981668 | MN969288 | KF296451 |
| *P*. *potchefstroomense* | CMV 011I2 ^T^ | MK450727 | MK451229 | MK451662 | MK450865 |
| *P*. *pulvillorum* | CBS 280.39 ^T^ | AF178517 | GU981670 | MN969289 | KF296452 |
| *P*. *raperi* | CBS 281.58 ^T^ | AF033433 | GU981622 | MN969291 | KF296453 |
| *P*. *reticulisporum* | CBS 122.68 ^T^ | AF033437 | MN969394 | MN969293 | KF296454 |
| *P*. *rolfsii* | CBS 368.48 ^T^ | JN617705 | GU981667 | MN969294 | KF296455 |
| *P*. *rotoruae* | CBS 145838 ^T^ | MN315103 | MN315104 | MN315102 | MT240842 |
| *P*. *rubriannulatum* | CGMCC 3.18804 ^T^ | KY495029 | KY495138 | MN969336 | KY495080 |
| *P*. *setosum* | CBS 144865 ^T^ | KT852579 | MF184995 | MH105905 | – |
| *P*. *siccitolerans* | FMR 17381 ^T^ | LR655193 | LR655197 | LR655201 | LR655205 |
| *P*. *silvertonense* | CMV 007E3 ^T^ | MK450731 | MK451122 | MK451666 | MK450869 |
| *P*. *simplicissimum* | CBS 372.48 ^T^ | GU981588 | GU981632 | MN969297 | JN121507 |
| *P*. *singorense* | CBS 138214 ^T^ | KJ775674 | KJ775167 | KJ775403 | MN969138 |
| *P*. *skrjabinii* | CBS 439.75 ^T^ | GU981576 | GU981626 | MN969299 | EU427252 |
| *P*. *soli* | KUMCC 18-0202 ^T^ | MT152337 | MT161681 | MT178249 | MT384372 |
| *P*. *soliforme* | CGMCC 3.18806 ^T^ | KY495038 | KY495147 | MN969337 | KY495047 |
| *P*. *soosanum* | CCF 3778 ^T^ | FJ430745 | FM865811 | LT970913 | LT797561 |
| *P*. *spinuliferum* | CGMCC 3.18807 ^T^ | KY495040 | KY495149 | MN969338 | KY495090 |
| *P*. *stangiae* | URM 8347 ^T^ | MW648590 | MW646388 | MW646390 | MW646392 |
| *P*. *stolkiae* ^OUT^ | CBS 315.67 ^T^ | AF033444 | JN617717 | AF481135 | JN121488 |
| *P*. *subfuscum* | CBS 147455 ^T^ | MT949907 | MT957412 | MT957454 | MT957480 |
| *P*. *subrubescens* | CBS 132785 ^T^ | KC346350 | KC346327 | KC346330 | KC346306 |
| *P*. *subrutilans* | CGMCC 3.25174 ^T^ | OQ870816 | OR051137 | OR051314 | OR051479 |
| *P*. *svalbardense* | CBS 122416 ^T^ | GU981603 | DQ486644 | KC346338 | KF296457 |
| *P*. *taii* | CGMCC 3.25176 ^T^ | OQ870778 | OR051170 | OR051347 | OR051496 |
| *P*. *tanzanicum* | CBS 140968 ^T^ | KT887841 | KT887802 | KT887763 | MN969183 |
| *P*. *tengii* | CGMCC 3.25179 ^T^ | OQ870735 | OR051120 | OR051297 | OR051465 |
| *P*. *terrarumae* | CBS 131811 ^T^ | MN431397 | KX650295 | MN969323 | MN969185 |
| *P*. *uruguayense* | CBS 143247 ^T^ | LT904729 | LT904699 | LT904698 | MN969200 |
| *P*. *uttarakhandense* | NFCCI 4808 ^T^ | MN967315 | MN972443 | MN972445 | MN972447 |
| *P*. *vanderhammenii* | CBS 126216 ^T^ | GU981574 | GU981647 | MN969308 | KF296458 |
| *P*. *vasconiae* | CBS 339.79 ^T^ | GU981599 | GU981653 | MN969309 | MN969144 |
| *P*. *vickeryae* | BRIP 72552a ^T^ | OP903479 | OP921966 | – | OP921965 |
| *P*. *viridissimum* | CGMCC 3.18796 ^T^ | KY495004 | KY495113 | MN969339 | KY495059 |
| *P*. *wandoense* | CNUFC-WT31-1 ^T^ | – | MK080564 | MK080566 | MK080562 |
| *P*. *wotroi* | CBS 118171 ^T^ | GU981591 | GU981637 | MN969313 | KF296460 |
| *P*. *yunnanense* | CGMCC 3.18794 ^T^ | KY494990 | KY495099 | MN969340 | KY495048 |
| *P*. *yuyongnianii* | CGMCC 3.25187 ^T^ | OQ870820 | OR051175 | OR051352 | OR051499 |
| *P*. *zhanjiangense* | CGMCC 3.25206 ^T^ | ON563149 | ON231294 | ON470837 | – |
| *P*. *zonatum* | CBS 992.72 ^T^ | GU981581 | GU981651 | MN969315 | KF296461 |

Fungal species obtained in this study are bold. Superscript “T” represents ex-type species. The superscript “OUT” represents the outgroup taxa. “–” represents the absence of sequence data in the GenBank database. Acronyms of culture collections: BRIP - the culture collection held in the Queensland Plant Pathology Herbarium, Queensland, Australia; CBS - the Westerdijk Fungal Biodiversity Institute, Utrecht, the Netherlands; CCDCA - Department of Food Science, Federal University of Lavras, Lavras - MG, Brazil; CCF - the Culture Collection of Fungi, Department of Botany, Charles University, Prague, Czech Republic; CGMCC - the China General Microbiological Culture Collection Center, Beijing, China; CMV - working collection housed at the PPRI, culture collection of the National Collections of Fungi, housed at the Agricultural Research Council - Plant Health and Protection (ARC), Roodeplaat, South Africa; CNUFC - the Chonnam National University Fungal Collection, Gwangju, South Korea; COAD - the Otávio de Almeida Drumond culture collection, Universidade Federal de Viçosa, Brazil; FMR - the fungal collection of the Faculty of Medicine, Reus, Spain; IBT - the culture collection of the Technical University of Denmark, Lyngby, Denmark; KUMCC - the Kunming Institute of Botany Culture Collection, Yunnan, China; MBSZU - the Culture Collection of Microbial Shenzhen University, Shenzhen, China; NFCCI - the National Fungal Culture Collection of India, Pune, India; URM - the Father Camille Torrend Herbarium, Pernambuco, Brazil.

**Table S3** GenBank accession numbers of taxa in Xylariomycetidae used in multi-genes phylogenetic analysis.

| **Species** | **Strain numbers** | **ITS** | **LSU** | ***RPB2*** | ***TUB*** |
| --- | --- | --- | --- | --- | --- |
| *Achaetomium macrosporum* ^OUT^ | CBS 532.94 | KX976574 | KX976699 | KX976797 | KX976915 |
| *Alloanthostomella rubicola* | MFLUCC 16-0479 | KX533455 | KX533456 | – | – |
| *Amphisphaeria hydei* | CMUB 40016 ^T^ | OR507141 | OR507154 | OR504417 | OR519975 |
| *Amphisphaeria sambuci* | CBS 131707 ^T^ | KT949904 | KT949904 | MH554911 | MH704632 |
| *Amphisphaeria thailandica* | MFLU 18-079 ^T^ | MH971225 | MH971235 | MK033640 | MK033639 |
| *Anthostomella formosa* | MFLUCC 14-0170 | MW240652 | KP340544 | KP340531 | MW820917 |
| *Anthostomella helicofissa* | MFLUCC 14-0173 ^T^ | KP297406 | KP297406 | KP340534 | KP406617 |
| *Anthostomelloides krabiensis* | MFLUCC 15-0678 ^T^ | KX305927 | KX305928 | KX305929 | – |
| *Apiospora camelliae-sinensis* | CGMCC 38333 ^T^ | KY494704 | KY494780 | – | KY705173 |
| *Apiospora dichotomanthis* | CGMCC 38332 ^T^ | KY494697 | KY494773 | – | KY705167 |
| *Apiospora guiyangensis* | HKAS 102403 ^T^ | MW240647 | MW240577 | MW658634 | MW775604 |
| *Apiospora sichuanensis* | HKAS 107008 ^T^ | MW240648 | MW240578 | MW658635 | MW775605 |
| *Appendicospora hongkongensis* | HKAS 107015 | MW240651 | MW240581 | MW658638 | MW775609 |
| *Bagadiella lunata* | CBS 124762 ^T^ | GQ303269 | GQ303300 | – | – |
| *Barrmaelia macrospora* | CBS 142768 ^T^ | KC774566 | KC774566 | MF488995 | MF489014 |
| *Barrmaelia rhamnicola* | CBS 142772 ^T^ | MF488990 | MF488990 | MF488999 | MF489018 |
| *Beltrania rhombica* | CBS 123.58 ^T^ | MH857718 | MH869260 | MH554899 | MH704631 |
| *Beltraniella endiandrae* | CBS 137976 ^T^ | KJ869128 | KJ869185 | – | – |
| *Beltraniopsis longiconidiophora* | MFLUCC 17-2139 ^T^ | MF580249 | MF580256 | – | – |
| *Bicellulospora elaeidis* | CGMCC 3.24962 ^T^ | OR253161 | OR253319 | – | OR266104 |
| *Biscogniauxia nummularia* | MUCL 51395 ^T^ | KY610382 | KY610427 | KY624236 | KX271241 |
| *Cainia graminis* | CBS 136.62 ^T^ | KR092793 | AF431949 | – | – |
| *Castanediella acaciae* | CBS 139896 ^T^ | KR476728 | KR476763 | – | – |
| *Castanediella brevis* | KUMCC 18-0132 ^T^ | MH806361 | MH806358 | – | – |
| *Castanediella eucalypticola* | CBS 141317 ^T^ | KX228266 | KX228317 | – | KX228382 |
| *Castanediella malaysiana* | CPC 24918 ^T^ | KX306752 | KX306781 | – | – |
| *Castellaniomyces rosae* | MFLUCC 15-0536 ^T^ | MF614127 | MF614130 | – | – |
| *Catenuliconidia uniseptata* | GZCC 20-0036 | MK804515 | MK804516 | MK828513 | – |
| *Chaetomium elatum* ^OUT^ | CBS 374.66 | KC109758 | KC109758 | KF001820 | KC109776 |
| *Clypeophysalospora latitans* | CBS 141463 ^T^ | KX820250 | KX820261 | – | – |
| *Collodiscula fangjingshanensis* | GZUH 0109 ^T^ | KR002590 | KR002591 | KR002592 | KR002589 |
| *Coniocessia anandra* | CBS 125766 ^T^ | MH863747 | MH875215 | – | – |
| *Cylindrium aeruginosum* | CBS 693.83 | KM231854 | KM231734 | KM232430 | KM232124 |
| *Cylindrium algarvense* | CBS 124770 ^T^ | MH863409 | MH874925 | – | – |
| *Cylindrium grande* | CBS 145655 ^T^ | MK876384 | MK876425 | MK876481 | MK876502 |
| *Cylindrium purgamentum* | CPC 29580 ^T^ | KY173435 | KY173525 | – | – |
| *Dactylaria acaciae* | CPC 29771 ^T^ | KY173400 | KY173493 | – | – |
| *Dactylaria retrophylli* | CBS 148271 ^T^ | ON811489 | ON811548 | – | – |
| *Daldinia andina* | CBS 114736 ^T^ | AM749918 | KY610430 | KY624239 | KC977259 |
| *Daldinia dennisii* | CBS 114741 ^T^ | JX658477 | KY610435 | KY624244 | KC977262 |
| *Delonicicola siamense* | MFLUCC 15-0670 ^T^ | MF167586 | MF158345 | MF158346 | – |
| *Diatrype disciformis* | CBS 197.49 | – | DQ470964 | DQ470915 | – |
| *Endocalyx indumentum* | JCM 5171 ^T^ | MZ313153 | MZ313161 | – | – |
| *Entosordaria perfidiosa* | CBS 142773 ^T^ | MF488993 | MF488993 | MF489003 | MF489021 |
| *Entosordaria quercina* | CBS 142774 ^T^ | MF488994 | MF488994 | MF489004 | MF489022 |
| *Eutypa cerasi* | GMBC0049 | MW797105 | MW797049 | MW814895 | MW814877 |
| *Graphostroma platystomum* | CBS 270.87 ^T^ | JX658535 | DQ836906 | KY624296 | HG934108 |
| *Gyrothrix eucalypti* | CBS 146023 ^T^ | MN562109 | MN567617 | ON399346 | – |
| *Hansfordia pruni* | CBS 194.56 ^T^ | MK442585 | MH869122 | KU684307 | – |
| *Hansfordia pulvinata* | CBS 144422 | MK442587 | MK442527 | – | – |
| *Hyponectria buxi* | UME 314 | – | AY083834 | – | – |
| *Hypoxylon petriniae* | CBS 114746 ^T^ | KY610405 | KY610491 | KY624279 | KX271274 |
| *Induratia apiospora* | ATCC 60639 ^T^ | OP862879 | OP862881 | OP879469 | OP879468 |
| *Iodosphaeria honghense* | MFLU 19-0719 ^T^ | MK737501 | MK722172 | MK791287 | – |
| *Iodosphaeria tongrenensis* | GZUH0109 ^T^ | KR095282 | KR095283 | – | – |
| *Leiosphaerella lycopodina* | CBS 125717 | MH863717 | – | – | – |
| *Leiosphaerella praeclara* | CBS 125586 | JF440976 | JF440976 | – | – |
| *Leptosillia muelleri* | CBS 143628 ^T^ | MK527857 | MK527857 | MK523290 | MK523347 |
| *Lopadostoma dryophilum* | CBS 133213 ^T^ | KC774570 | KC774570 | KC774526 | MF489023 |
| *Lopadostoma turgidum* | CBS 133207 ^T^ | KC774618 | KC774618 | KC774563 | MF489024 |
| *Melanographium phoenicis* | MFLUCC 18-1481 ^T^ | MN482677 | MN482678 | – | – |
| *Melanographium smilacis* | MFLU 21-0075 ^T^ | MZ538514 | MZ538548 | – | – |
| *Melogramma campylosporum* | MFLU 18-0778 | MW240646 | MW240576 | MW658633 | MW775603 |
| *Melogramma campylosporum* | MFLU 17-0348 | MW240645 | MW240575 | MW658632 | MW775602 |
| *Microdochium phragmitis* | CBS 285.71 ^T^ | KP859013 | KP858949 | KP859122 | KP859077 |
| *Microdochium seminicola* | CBS 139951 ^T^ | KP859038 | MH878672 | KP859147 | KP859101 |
| *Nemania abortiva* | BISH 467 ^T^ | GU292816 | – | GQ844768 | GQ470219 |
| *Neoamphisphaeria hyalinospora* | MFLU 19-2131 ^T^ | MW240649 | MW240579 | MW658636 | MW775607 |
| *Neoamphisphaeria hyalinospora* | HKAS 106988 | MW240650 | MW240580 | MW658637 | MW775608 |
| *Neoanthostomella bambusicola* | MFLU 18-0796 ^T^ | MW240657 | MW240587 | MW658641 | MW775610 |
| *Neoanthostomella fici* | MFLU 19-2765 ^T^ | MW114390 | MW114445 | MW177711 | – |
| *Neoarthrinium moseri* | CBS 164.80 ^T^ | LN850995 | LN851049 | – | LN851154 |
| *Neoarthrinium trachycarpi* | CFCC 53039 ^T^ | MK301099 | – | – | MK303395 |
| *Neobarrmaelia hyphaenes* | CPC 40101 = CBS 148304 ^T^ | ON811507 | ON811566 | ON803546 | ON803594 |
| *Neobarrmaelia serenoae* | CPC 37572 = CBS 146017 ^T^ | MT223781 | MT223876 | – | MT223730 |
| *Neogyrothrix oleae* | CBS 146068 | MN562137 | MN567644 | – | – |
| *Neogyrothrix oleae* | CBS 146069 ^T^ | MN562136 | MN567643 | – | – |
| *Neoleptodontidium aciculare* | CBS 123.86 ^T^ | MH861931 | MH873620 | – | – |
| *Neoleptodontidium aquaticum* | CBS 149455 = CPC 42868 ^T^ | OQ990116 | OQ990067 | – | – |
| *Neophysalospora eucalypti* | CBS 138864 ^T^ | KP004462 | MH878627 | – | – |
| *Nigropunctata nigrocircularis* | MFLU 19-2130 ^T^ | MW240661 | MW240591 | – | MW775612 |
| *Nigropunctata saccata* | MFLU 19-2144 ^T^ | MW240663 | MW240593 | MW658645 | MW775613 |
| *Nigrospora gorlenkoana* | CBS 480.73 ^T^ | KX986048 | KX986109 | – | KY019456 |
| *Nigrospora musae* | CBS 319.34 ^T^ | KX986076 | KX986110 | – | KY019455 |
| *Oxydothis metroxylonicola* | MFLUCC 15-0281 ^T^ | KY206774 | KY206763 | – | – |
| *Oxydothis palmicola* | MFLUCC 15-0806 ^T^ | KY206776 | KY206765 | – | – |
| *Oxydothis rhapidicola* | MFLUCC 14-0616 ^T^ | – | KY206766 | – | – |
| *Parabartalinia lateralis* | CBS 399.71 ^T^ | MH554043 | MH554256 | MH554954 | MH554719 |
| *Paraphysalospora eucalypti* | CBS 143177 ^T^ | MG386038 | MG386091 | – | – |
| *Parapleurotheciopsis caespitosa* | CBS 519.93 ^T^ | MH862437 | MH874086 | – | – |
| *Phlogicylindrium eucalyptorum* | CBS 120221 | EU040223 | MH554204 | MH554894 | MH704635 |
| *Phlogicylindrium uniforme* | CBS 131312 ^T^ | JQ044426 | JQ044445 | MH554910 | MH704634 |
| *Pidoplithchkoviella terricola* | CBS 180.77 ^T^ | MH861046 | AF096197 | – | – |
| *Pirozynskiomyces sinensis* | UAMH 11913 ^T^ | KY994106 | KY994107 | – | – |
| *Plectosphaera eucalypti* | CBS 120063 ^T^ | DQ923538 | DQ923538 | – | – |
| *Pseudoanthostomella pini-nigrae* | MFLUCC 16-0478 ^T^ | KX533453 | KX533454 | KX789492 | – |
| *Pseudoanthostomella senecionicola* | MFLUCC 15-0013 ^T^ | MW240674 | MW240604 | MW658653 | MW820913 |
| ***Pseudoleptodontidium chiangmaiense*** | **MBSZU 25-005** **^T^** | **PV036872** | **PV036873** | **PV054947** | **PV054946** |
| *Pseudosporidesmium knawiae* | CBS 123529 ^T^ | MH863299 | MH874823 | – | – |
| *Pseudosporidesmium lambertiae* | CBS 143169 ^T^ | MG386034 | MG386087 | – | – |
| *Pseudotruncatella arezzoensis* | MFLUCC 14-0988 ^T^ | MG192320 | MG192317 | – | – |
| *Pseudotruncatella bolusanthi* | CBS 145532 ^T^ | MK876407 | MK876448 | – | – |
| *Seimatosporium rosae* | MFLUCC 14-0621 ^T^ | LT853105 | KT198727 | LT853153 | LT853253 |
| *Seiridium cupressi* | CBS 224.55 ^T^ | LT853083 | MH868999 | LT853131 | LT853230 |
| *Seiridium marginatum* | CBS 140403 ^T^ | KT949914 | MH878679 | LT853149 | LT853249 |
| *Sordaria fimicola* ^OUT^ | CBS 723.96 | MH862606 | MH874231 | DQ368647 | – |
| *Strickeria kochii* | CBS 140411 ^T^ | KT949918 | MH878680 | MH554920 | MH554679 |
| *Subanthostomella palmae* | SNT317 ^T^ | PP592484 | PP621114 | PP780202 | PP816199 |
| *Subanthostomella palmae* | SNT325 | PP592485 | PP621115 | PP780203 | PP816200 |
| *Subsessila turbinata* | MFLUCC 15-0831 ^T^ | KX762288 | KX762289 | – | – |
| *Synnemadiella eucalypti* | CPC 27637 ^T^ | KY173467 | KY173556 | – | – |
| *Synnemapestaloides juniperi* | CBS 477.77 ^T^ | MH554053 | MH554266 | MH554966 | MH554729 |
| *Truncatella angustata* | CBS 144025 ^T^ | MH554112 | MH554318 | MH555021 | MH554785 |
| *Vamsapriya bambusicola* | MFLUCC 11-0477 ^T^ | KM462835 | KM462836 | KM462834 | KM462833 |
| *Vamsapriya breviconidiophora* | MFLUCC 14-0436 ^T^ | MF621584 | MF621588 | – | – |
| *Vialaea insculpta* | DAOM 240860 | JX123570 | – | – | – |
| *Vialaea insculpta* | DAOM 240257 | JX139726 | JX139726 | – | – |
| *Xenoanthostomella parvispora* | CMUB 40019 ^T^ | OR507143 | OR507156 | OR504419 | OR519977 |
| *Xylaria bambusicola* | WSP 205 ^T^ | EF026123 | – | GQ844802 | AY951762 |

Fungal species obtained in this study are bold. Superscript “T” represents ex-type species. The superscript “OUT” represents the outgroup taxa. “–” represents the absence of sequence data in the GenBank database. Acronyms of culture collections: ATCC - the American Type Culture Collection, Manassas, USA; BISH: Bishop Museum, Honolulu, USA; CBS - the Westerdijk Fungal Biodiversity Institute, Utrecht, the Netherlands; CFCC - the China Forestry Culture Collection Center, Research Institute of Forest Ecology, Environment and Protection, Beijing, China; CGMCC - the China General Microbiological Culture Collection Center, Beijing, China

CMUB - the Chiang Mai University Herbarium, Chiang Mai, Thailand; CPC - the Culture collection of Pedro Crous, housed at CBS, Utrecht, the Netherlands; DAOM - the Canadian National Mycological Herbarium, Ontario, Canada; GMBC - the Guizhou Medical University Collection Centre, Guizhou, China; GZCC - Guizhou Academy of Agricultural Sciences, Guizhou, China; GZUH - Guizhou University, Guiyang, China; HKAS - Herbarium of Cryptogams Kunming Institute of Botany Academia Sinica, China; JCM - Japan Collection of Microorganisms, Ibaraki, Japan; KUMCC - the Kunming Institute of Botany Culture Collection, Yunnan, China; MBSZU - the Culture Collection of Microbial Shenzhen University, Shenzhen, China; MFLU, MFLUCC - Mae Fah Luang University, Chiang Rai, Thailand; UAMH - the UAMH Centre for Global Microfungal Biodiversity at University of Toronto, Toronto, Canada; WSP - Washington State University, Pullman, USA.
